# Isolation of Carbene‐Stabilized Arsenic Monophosphide [AsP] and its Radical Cation [AsP]^+.^ and Dication [AsP]^2+^

**DOI:** 10.1002/chem.201903795

**Published:** 2019-09-13

**Authors:** Adinarayana Doddi, Dirk Bockfeld, Marc‐Kevin Zaretzke, Thomas Bannenberg, Matthias Tamm

**Affiliations:** ^1^ Department of Chemical Sciences Indian Institute of Science Education and Research Berhampur, Transit Campus, Govt. ITI Building Engineering School Road, Ganjam, Berhampur 760010 Odisha India; ^2^ Institut für Anorganische und Analytische Chemie Technische Universität Braunschweig Hagenring 30 38106 Braunschweig Germany

**Keywords:** arsenic, arsenic monophosphide, main group elements, N-heterocyclic carbenes, phosphorus

## Abstract

Arsenic monophosphide (AsP) species supported by two different N‐heterocyclic carbenes were prepared by reaction of (IDipp)PSiMe_3_ (**1**) (IDipp=1,3‐bis(2,6‐diisopropylphenyl)imidazolin‐2‐ylidene) with (IMes)AsCl_3_ (**2**) (IMes=1,3‐bis(2,4,6‐trimethylphenyl)imidazolin‐2‐ylidene) to afford the dichloride [(IMes)As(Cl)P(IDipp)]Cl (**3**), which upon reduction with KC_8_ furnished heteroleptic [(IMes)AsP(IDipp)] (**4**). The corresponding mono‐ and dications [(IMes)AsP(IDipp)][PF_6_], [**5**]PF_**6**_, and [(IMes)AsP(IDipp)][GaCl_4_]_2,_ [**6**][GaCl_4_]_2_, respectively, were prepared by one‐electron oxidation of **4** with ferrocenium hexafluorophosphate, [Fc]PF_6,_ or by chloride abstraction from **3** with two equivalents of GaCl_3_, respectively. Compounds **4**–**6** represent rare examples of heterodiatiomic interpnictogen compounds, and X‐ray crystal structure determinations together with density functional theory (DFT) calculations reveal a consecutive shortening of the As−P bond lengths and increasing bond order, in agreement with the presence of an arsenic–phosphorus single bond in **4** and a double bond in **6** ^2+^. The EPR signal of the cationic radical [**5**]^+.^ indicates a symmetric spin distribution on the AsP moiety through strong hyperfine coupling with the ^75^As and ^31^P nuclei.

## Graphical abstract

**Arsenic monophosphide** [AsP] is stabilized by N‐heterocyclic carbenes in [(IMes)AsP(IDipp)], which together with the corresponding [AsP]^+.^ and [AsP]^2+^ systems represent rare examples of heterodiatomic dicarbene complexes. One‐ and two‐electron oxidation leads to a consecutive shortening of the As−P bond length with an increase from single to double bond character, with the intermediate radical cation showing a symmetric spin distribution on the AsP moiety.

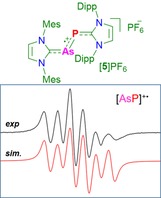

Carbene stabilization of diatomic allotropes has been among the most successful applications of N‐heterocyclic carbenes (NHC) in main‐group element chemistry,^1^ with the homodinuclear systems [(IDipp)E_2_(IDipp)] (E=B,^2^ Si,^3^ Ge,^4^ Sn,^5^ P,^6^ As,^7^ IDipp=bis(2,6‐diisopropylphenyl)imidazolin‐2‐ylidene) representing the most prominent examples. In contrast, heterodinuclear species are rare, and to the best of our knowledge, the heteroleptic carbene‐stabilized “phosphorus mononitride” **A** and its radical cation (PN^+**⋅**^) represent the only compounds, in which a diatomic moiety consisting of two different p‐block elements is supported by two carbene ligands (Figure 1).^8^ The controlled synthesis of such species requires a modular approach for assembling the two different (NHC)E sides, and we have recently established such a protocol successfully for the preparation of the heteroleptic dicarbene–diphosphorus species [(IMes)P_2_(IDipp)] (**B**, IMes=bis(2,4,6‐trimethylphenyl)imidazolin‐2‐ylidene),^9^ which represents a hybrid of the previously reported homoleptic congeners [(IMes)P_2_(IMes)] (**C**) and [(IDipp)P_2_(IDipp)] (**D**).^6^ Moreover, in a similar manner as described for **D** and also for the analogous diarsenic compound [(IDipp)As_2_(IDipp)] (**E**),^10^, ^11^ the corresponding radical cation [(IMes)P_2_(IDipp)]^+.^ and dication [(IMes)P_2_(IDipp)]^2+^ were also prepared and structurally characterized. Notably, the heteroleptic nature of this system allowed for establishing phosphorus–phosphorus coupling in the diamagnetic neutral and dicationic forms.^9^

**Figure 1:**
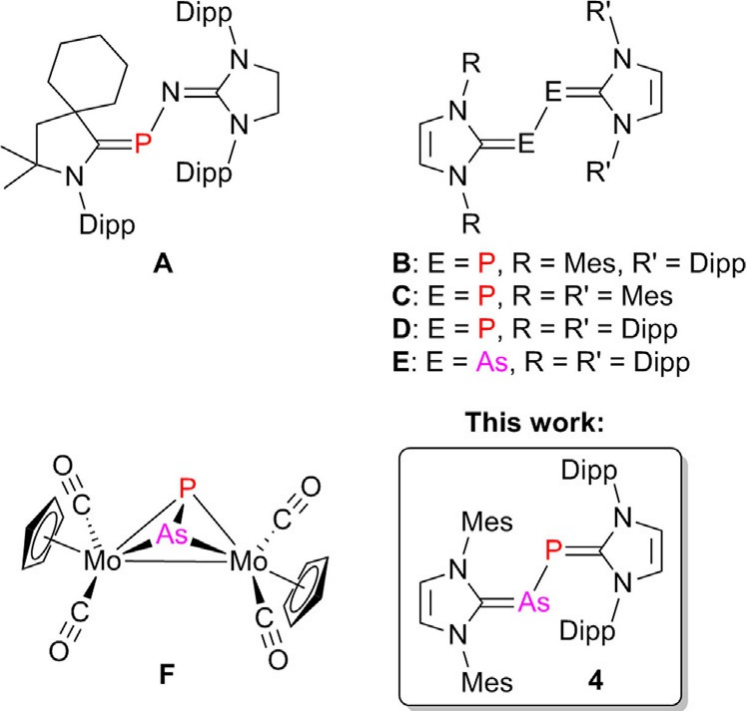
Examples of homo‐ and heterodinuclear dipnictogen compounds (Dipp=2,6‐diisopropylphenyl, Mes=2,4,6‐trimethylphenyl).

We reasoned that a similar modular approach could give access to heterodiatomic Group 15 species and therefore aimed at the isolation of carbene‐stabilized arsenic monophosphide [(IMes)AsP(IDipp)] (**4**). Like homodinuclear P_2_ and As_2_, the heterodinuclar AsP molecule was studied spectroscopically in the gas phase^12^ or in solid neon matrix,^13^ and a dissociation energy of 429.7(12.6) kJ mol^−1^ was determined by mass spectrometry. This energy falls between the values of 485.8(0.4) and 379.1(9.6) kJ mol^−1^ derived for P_2_ and As_2_, respectively.^14^ In the solid state, arsenic monophosphide was found to crystallize in the orthorhombic crystal system,^15^ and this black arsenic–phosphorus form has recently emerged as a promising candidate for two‐dimensional electronic materials application.^16^ AsP and other unsupported heterodiatomic Group 15 ligands were also stabilized in transition‐metal complexes such as [Cp_2_Mo_2_(CO)_4_(μ‐η^2^:η^2^‐PAs)] (**F**), which contains a Mo_2_AsP tetrahedrane core.^17^, ^18^ Tetrahedral AsP_3_ is another interpnictide molecule that has been employed as a ligand in coordination chemistry.^19^, ^20^

The N‐heterocyclic carbene–trimethylsilylphosphinidene adduct (IDipp)PSiMe_3_ (**1**) served as an excellent synthon for the transfer of the (IDipp)P moiety by reaction with main‐group element and transition‐metal halides.^9^, ^21^ Likewise, the equimolar reaction of **1** with the NHC adduct of arsenic trichloride (IMes)AsCl_3_ (**2**) at room temperature in toluene afforded the dichloro species **3** which was isolated as an orange solid in 79 % yield and in pure form according to elemental analysis (Scheme 1). The ^31^P NMR spectrum (Figure S5, Supporting Information) of **3** in CD_2_Cl_2,_ however, showed four different signals at 132.2, 20.3, 16.5, and 1.8 ppm at room temperature, indicating the presence of isomers with different chlorine binding modes (Scheme S1). Single crystals of **3**⋅3 CH_2_Cl_2_ were isolated from dichloromethane/hexane solution, and X‐ray diffraction analysis revealed the formation of the ionic compound [(IMes)As(Cl)P(IDipp)]Cl containing a chloride counterion and one chlorine atom bound to the arsenic atom (Figure 2). The As−P bond length is 2.2152(6) Å, which is almost identical with the As−P distances reported for the cationic *C*_2_‐symmetric compound [(IDipp)PAsP(IDipp)]Cl (2.203(1) Å)^22^ and for the bimetallic AsP complex **F** (2.2324(13) Å).^17^ Slightly longer bonds were found for other related NHC arsenic–phosphorus systems, for example 2.3133(4) Å in [(SIMes)PAs(*t*Bu)_2_]^23^ or 2.3552(6) Å in the IDipp adduct of a transient phosphino–arsinidene (SIMes=1,3‐bis(2,4,6‐trimethylphenyl)imidazolidin‐2‐ylidene).^24^

**Scheme 1:**
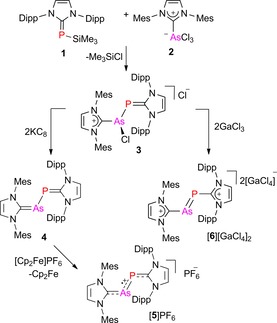
Preparation of heteroleptic N‐heterocyclic carbene‐supported AsP species.

**Figure 2:**
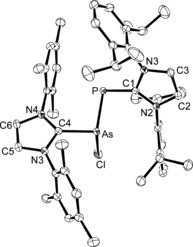
ORTEP diagram of the cationic part in [(IMes)As(Cl)P(IDipp)]Cl (**3**) with thermal displacement parameters drawn at 50 % probability level. Hydrogen atoms are omitted for clarity. Selected bond lengths [Å] and angles [°]: As−P 2.2152(6), As−C4 1.9848(16), P−C1 1.8132(17); C1‐P‐As 93.64(6), C4‐As‐P 97.58(5), N3‐C4‐N4 106.28(14), N2‐C1‐N1 105.76(14).

Reduction of **3** with two equivalents of potassium graphite (KC_8_) in THF afforded [(IMes)AsP(IDipp)] (**4**) which was isolated as a dark red solid in 37 % yield (Scheme 1). The ^31^P NMR spectrum (in C_6_D_6_, Figure S8, Supporting Information) showed a broad signal at −60.6 ppm, which lies between the chemical shifts established for the homoleptic diphosphorus species [(IDipp)P_2_(IDipp)] (**B**, −52.4 ppm) and [(IMes)P_2_(IMes)] (−73.6 ppm),^6^ whereas the heteroleptic congener [(IMes)P_2_(IDipp)] afforded two doublets with similar chemical shifts at −63.1 and −59.4 ppm (^1^ *J*_PP_=249 Hz).^9^ The ^1^H NMR spectrum of **4** at room temperature shows the signals expected for the IMes and IDipp ligands, for instance at 5.83 and 6.00 ppm for the backbone imidazole CH hydrogen atoms; these signals are broadened, indicating slightly hindered rotation around the E−C bonds (E=P, As). In the ^13^C NMR spectrum, two characteristic doublets are found for the carbene carbon atoms at 169.0 ppm (^1^ *J*_PC_=112 Hz) and 171.0 ppm (^2^ *J*_PC_=17 Hz).

The molecular structure of **4** was established by X‐ray diffraction analysis (Figures 3 and 5), confirming the expected *trans*‐bent geometry with a slightly twisted, but almost coplanar orientation of the imidazole rings towards the central AsP unit as indicated by torsion angles of 162.25(13)° (C1‐P‐As‐C4), 179.38(19)° (N2‐C1‐P‐As), and 172.91(18)° (N3‐C4‐P‐As). The As−P bond length is 2.3149(8) Å,^25^ which is consistent with an As−P single bond (2.32 Å)^26^ and intermediate between the P−P distances in the corresponding diphosphorus systems, specifically 2.1897(4) (**B**),^9^ 2.1897(11) (**C**),^6^ and 2.2052(10) Å (**D**),^6^ as well as the As−As distance of 2.442(1) Å in the diarsenic compound **E**.^7^ A virtually identical As−P bond length of 2.3133(4) Å was found for [(SIMes)PAs(*t*Bu)_2_].^23^ It also falls in the range established for other As−P single bonds, as for instance in cyclodiarsadiphosphanes,^27^ or in complexes containing the tetrahedral AsP_3_ ligand.^19^, ^20^ The phosphorus–carbon and arsenic–carbon bond lengths of 1.750(3) (P−C1) and 1.884(2) Å (As−C4) are well within the range established for carbene–pnictinidene adducts, for example 1.763(6) and 1.899(3) Å in (IMes)EPh (E=P, As)^28^ or 1.752(1) and 1.883(2) Å in (IDipp)EH (E=P, As),^22^, ^29^ in agreement with the presence of polarized P−C and As−C double bonds.

**Figure 3:**
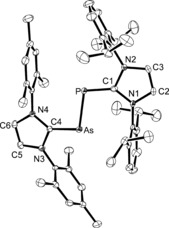
ORTEP diagram of [(IMes)AsP(IDipp)] (**4**) with the thermal displacement parameters drawn at 50 % probability level. Hydrogen atoms are omitted for clarity. Selected bond lengths [Å] and angles [°]: As−P 2.3149(8), As−C4 1.884(2), P−C1 1.750(3); As‐P‐C1 101.61(9), P‐As‐C4 100.40(8), N3‐C4‐N4 103.91(19), N1‐C1‐N2 103.3(2).

It was previously demonstrated that the dicarbene‐supported P_2_ and As_2_ species **D** and **E** can be oxidized stepwise to the respective radical cation and dications.^10^, ^11^ Accordingly, treatment of **4** with one equivalent of ferrocenium hexafluorophosphate ([Fc]PF_6_) in THF afforded [(IMes)AsP(IDipp)]PF_6_, [**5**]PF_6_, as a dark green solid in 38 % yield (Scheme 1). Compound [**5**]PF_6_ represents a rare example of a carbene‐stabilized main‐group radical species containing two different p‐block elements,^30^ and it was fully characterized by elemental analysis, EPR spectroscopy, and X‐ray crystal‐structure determination. The X‐band EPR spectrum of a CH_2_Cl_2_ solution at room temperature is shown in Figure 4 (left); it shows a multiplet with an isotropic *g* value of 2.0246 and hyperperfine coupling to the ^75^As (*I*=3/2 ) and ^31^P (*I*=1/2 ) nuclei. The simulation afforded coupling constants of *A*(^75^As)=21.7 G, *A*(^31^P)=42.2 G, and *A*(^14^N)=1.5 G (not resolved), in excellent agreement with the EPR spectrum recorded in situ for the arsaphosphene radical anion [RAsPR]^−**⋅**^ (R=2,6‐(Mes)_2_‐4‐MeC_6_H_2_), which afforded a similar “pseudo‐sextet” pattern with *A*(^75^As)=23 G and *A*(^31^P)=48 G.^31^ The calculated spin density is distributed symmetrically between the arsenic (0.39 *e*) and phosphorus atoms (0.31 *e*) with only little additional localization on each of the four nitrogen atoms (approx. 0.04 *e*, Figure 4, right).

**Figure 4:**
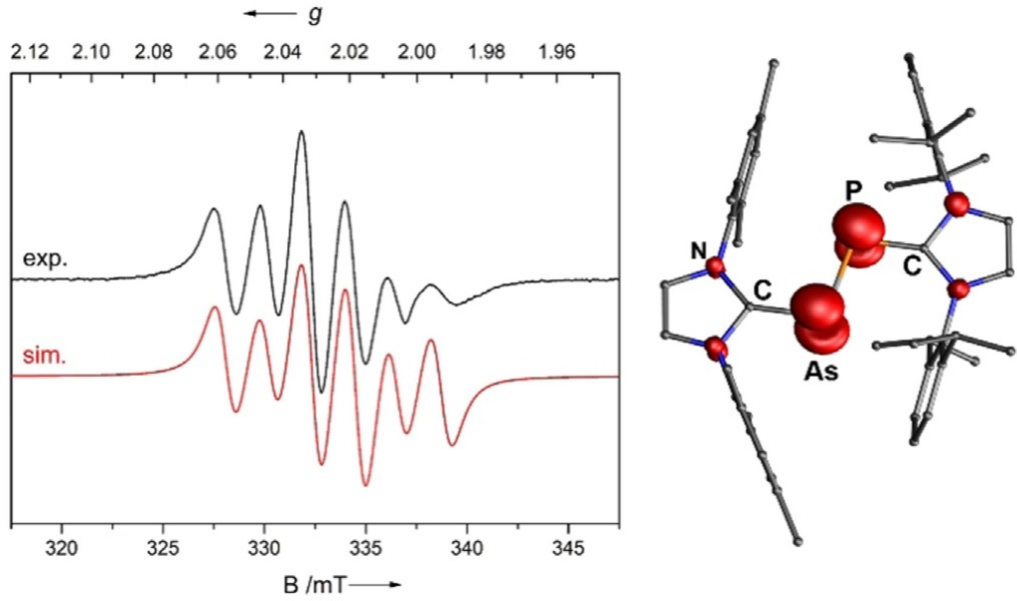
Left: Experimental (black) and simulated (red) EPR spectrum of [**5**]PF_6_ in CH_2_Cl_2_ (298 K, 9.448437 GHz). Right: Spin‐density distribution of radical [**5**]^+**⋅**^.

Further oxidation of **5** ^+**⋅**^, or two‐electron oxidation of **4**, furnished the dication [(IMes)AsP(IDipp)]^2+^ (**6** ^2+^) however, it is also conveniently accessible from **3** through abstraction of the two chloride ions with two equivalents of gallium trichloride. This reaction was performed by addition of GaCl_3_ to a THF solution of **3** at room temperature to afford [**6**][GaCl_4_]_2_ as a dark orange crystalline solid in 47 % yield after thorough washing with fluorobenzene and hexane (Scheme 1). The ^31^P NMR spectrum of [**6**][GaCl_4_]_2_ gives rise to a characteristic lowfield resonance at 475.5 ppm (Figure S10, Supporting Information), which is in good agreement with the chemical shifts reported for homoleptic [(IDipp)P_2_(IDipp)][OTf]_2_ (*δ*=452 ppm)^10^ and heteroleptic [(IMes)P_2_(IDipp)][GaCl_4_]_2_ (*δ*=438.5/440.1 ppm ^1^ *J*_PP_=543 Hz),^9^ whereas arsaphosphenes of the type RAs=PR′ (R, R′=aryl, alkyl) generally feature chemical shifts at even lower field (>500 ppm).^32^ It should be noted that the ^1^H and ^13^C NMR signals are sharper than those of **4**, revealing that rotation around the As−C and P−C bonds in **6** ^2+^ is faster on the NMR time scale, in agreement with a reduction of the corresponding bond orders (see below). The ^13^C NMR spectrum exhibits two doublets for the carbene carbon atoms at 152.2 (^1^ *J*_PC_=100 Hz) and 151.3 ppm (^2^ *J*_PC_=21 Hz).

The molecular structures of [**5**]PF_6_⋅CH_2_Cl_2_ and [**6**][GaCl_4_]_2_⋅2C_6_H_5_F were established by X‐ray diffraction analyses; [**5**]PF_6_⋅CH_2_Cl_2_ crystallizes with two independent molecules in the asymmetric unit, and ORTEP diagrams of all species are presented in the Supporting Information (Figures S22 and S23). Figure 5 shows an overlay of the structures of **4**, **5** ^+.^, and **6** ^2+^, revealing that the *trans*‐bent geometry and the nearly coplanar arrangement of the AsP unit and the imidazole planes in **4** is retained in the oxidized species. Upon oxidation, the As−P bond lengths decrease from 2.3149(8) in **4** to 2.2379(4)/2.2416(4) in **5** ^+.^ and 2.1610(8) Å in **6** ^2+^, which is accompanied by an elongation of the As−C and P−C bonds (Table 1). The As−P bond in radical **5** ^+.^ is slightly shorter than the corresponding bonds in the neutral radical [(IDipp)_2_(μ‐PAsP)] (2.256(1)/2.266(1) Å),^22^ whereas this bond length in **6** ^2+^ corresponds to an As=P double bond (2.16 Å).^25^ Consequently, similar, albeit slightly shorter As−P distances were found in arsaphosphenes of the type RAs=PR′,^32^, ^33^ such as 2.125(1) Å for R=CH(SiMe_3_) and R′=2,4,6‐*t*Bu_3_C_6_H_2_),^34^ 2.141(5) Å for R=R′=2,4,6‐*t*Bu_3_C_6_H_2_),^35^ and 2.134(2) Å for R=(2,4,6‐*i*Pr_3_C_6_H_2_)_2_C_6_H_3_, and R′=Mes.^36^ However, it should be noted that diarylarsaphosphenes just like the corresponding diphosphenes exhibit orthogonal orientations of the aryl groups towards the central dipnictene unit. A virtually identical As−P bond length of 2.161(1) Å was recently established for a cyclic *cis*‐arsaphosphene.^26^

**Figure 5:**
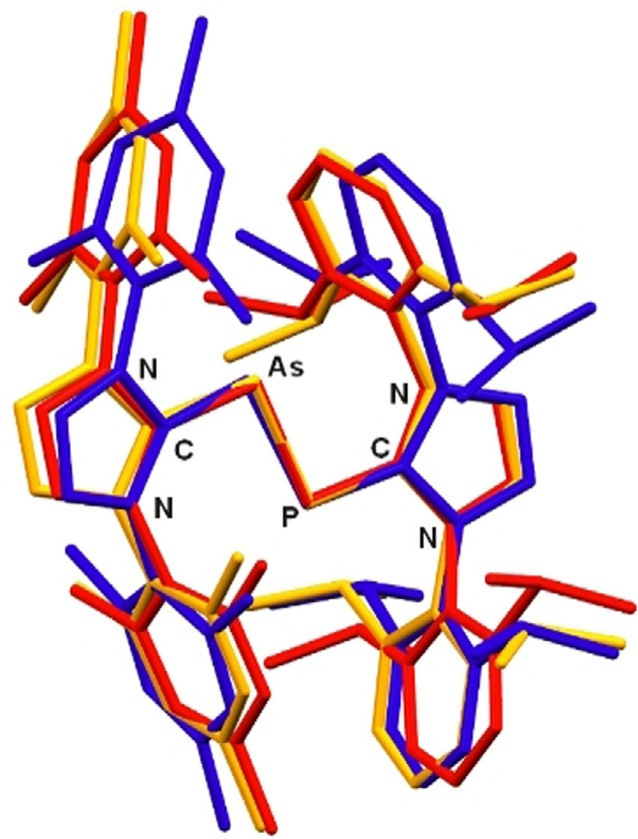
Overlay of the molecular structures of **4** (orange), **5** ^+.^ (blue), and **6** ^2+^ (red). Pertinent bond lengths are assembled in Table 1. Selected torsion angles [°] in **4**: C‐As‐P‐C 162.25(13), N‐C‐As‐P 172.91(18), N‐C‐P‐As 179.38(19); **5** ^+.^ (molecule 1/molecule 2): C‐As‐P‐C 171.04(7)/177.51(7), N‐C‐As‐P 169.35(13)/177.46(12), N‐C‐P‐As 177.89(11)/174.03(13); **6** ^2+^: C‐As‐P‐C 179.80(15), N‐C‐As‐P 173.9(3) and N‐C‐P‐As 175.2(2).

**Table 1:** Comparison of selected experimental (top) and calculated (bottom) bond lengths [Å] and associated Wiberg Bond Indices (WBI).

Comp.	As−P	WBI		As−C	WBI		P−C	WBI
**4**	2.3149(8)	1.02		1.883(2)	1.24		1.750(3)	1.35
	2.356			1.908			1.772	
**5** ^+**⋅**^	2.2379(4)/2.2416(4)	1.22		1.9377(15)/1.9425(15)	1.02		1.7995(15)/1.8000(15)	1.13
	2.288			1.957			1.810	
**6** ^2+^	2.1610(8)	1.63		1.959(3)	0.91		1.818(3)	1.02
	2.216			1.987			1.837	

To assess the bonding situation further in the arsenic–phosphorus species **4**, **5** ^+.^, and **6** ^2+^, their structures were optimized by the density functional theory (DFT) method at the B97‐D level of theory, followed by natural bond orbital (NBO) analysis. The computed structural parameters are in good agreement with those established by X‐ray diffraction analysis, although consistently longer As−P, As−C and P−C bond lengths were obtained (Table 1). The shortening of the As−P bonds with consecutive elongation of the As−C and P−C bonds in the series **4**, **5** ^+.^, and **6** ^2+^ is accurately reproduced, and the increase of the Wiberg bond index (WBI) from 1.02 in **4** to 1.22 in **5** ^+.^ and 1.63 in **6** ^2+^ corroborates the gradual increase of the As−P bond order from single to double‐bond character. This trend can be conveniently rationalized by analysis of the frontier molecular orbitals in **4**, **5** ^+.^, and **6** ^2+^ (Figure 6), revealing that the highest occupied molecular orbital in **4** represents mainly the antibonding π*(As−P) orbital, and stepwise oxidation with removal of electrons from this orbital will generate the singly occupied molecular orbital (SOMO) in **5** ^+.^ and lastly the lowest unoccupied molecular orbital (LUMO) in **6** ^2+^.

**Figure 6:**
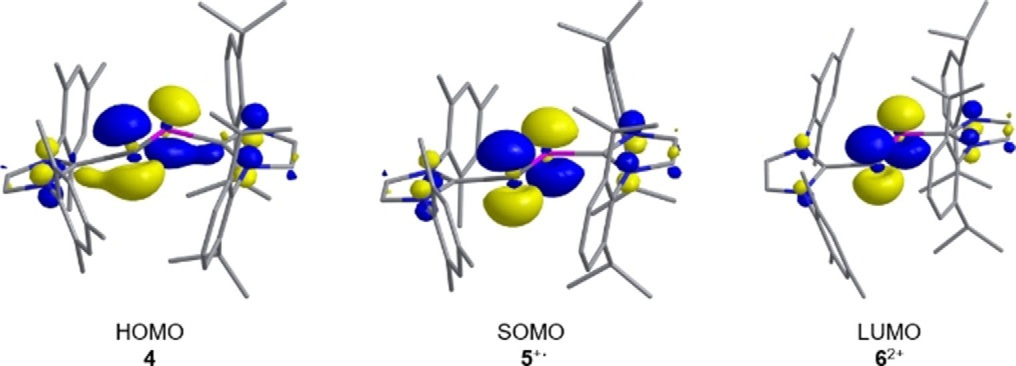
Frontier molecular orbitals in **4**, **5** ^+.^, and **6** ^2+^ with π*(As−P) character.

To the best of our knowledge, the arsenic monophosphide species [(IMes)AsP(IDipp)] (**4**), [(IMes)AsP(IDipp)]^+.^ (**5**), and [(IMes)AsP(IDipp)]^2+^ (**6**) represent the first system, in which N‐heterocyclic carbenes have been used for the stabilization of highly reactive heterodiatomic molecules containing two different heavier main‐group elements. The heteroleptic set of NHC ligands allowed to establish the solid‐state structures unequivocally by avoiding crystallographic disorder of the As and P atoms. The synthesis of this series built on a modular approach with (IDipp)PSiMe_3_ (**1**) serving as the synthon for the introduction of low‐valent phosphorus. With **1** and also the corresponding arsinidene adducts (NHC)AsSiMe_3_ at hand,^29^ numerous other heterodiatomic species of the type [(NHC)EE′(NHC)] (E=P, As; E′=Group 13, 14, 15 element) should be accessible.

Furthermore, the reactivity of [(IMes)AsP(IDipp)] (**4**) for example towards O_2_ and N_2_O to stabilize elusive arsenic–phosphorus oxide species,^37^ and its potential to release the AsP molecule for applications in transition‐metal chemistry and materials science will be investigated.^38^

## Conflict of interest

The authors declare no conflict of interest.

## Supporting information

As a service to our authors and readers, this journal provides supporting information supplied by the authors. Such materials are peer reviewed and may be re‐organized for online delivery, but are not copy‐edited or typeset. Technical support issues arising from supporting information (other than missing files) should be addressed to the authors.

Supplementary
