# Supplementary material for: Isolation of Carbene‐Stabilized Arsenic Monophosphide [AsP] and its Radical Cation [AsP]+. and Dication [AsP]2+
Source: Chemistry. 2019 Sep 13;25(57):13119–23. doi: 10.1002/chem.201903795 (PMC6856684; doi:10.1002/chem.201903795)
Supplement: Supplementary file 1 — Supplementary [file CHEM-25-13119-s001.pdf]

CHEMISTRY
A European Journal

Supporting Information

Isolation of Carbene-Stabilized Arsenic Monophosphide [AsP] and its Radical Cation $[AsP]^{+\cdot}$ and Dication $[AsP]^{2+}$

Adinarayana Doddi,*[a] Dirk Bockfeld,[b] Marc-Kevin Zaretzke,[b] Thomas Bannenberg,[b] and Matthias Tamm*[b]

chem_201903795_sm_miscellaneous_information.pdf

Table of Contents

A) General synthetic procedures and analytical methods

1) Preparation of (IMes)$AsCl_3$ (**2**)
2) Preparation of [(IMes)AsClP(IDipp)]Cl (**3**)
3) Preparation of [(IMes)AsP(IDipp)] (**4**)
4) Preparation of [(IMes)AsP(IDipp)]PF_6 [**5**]PF_6)
5) Preparation of [(IMes)AsP(IDipp)]$[GaCl_4]_2$ ([**6**]$[GaCl_4]_2$)

B) NMR and other spectra of compounds 2-6

1) ^1H, ^{13}C NMR spectra of (IMes)$AsCl_3$ (**2**)
2) ^1H, ^{13}C and ^{31}P NMR spectra of [(IMes)AsClP(IDipp)]Cl (**3**)
3) ^1H, ^{13}C and ^{31}P NMR spectra of [(IMes)AsP(IDipp)] (**4**)
4) ^1H, ^{13}C and ^{31}P NMR spectra of [(IMes)AsP(IDipp)]$[GaCl_4]_2$ ([**6**]$[GaCl_4]_2$)
5) Electron paramagnetic resonance (EPR) spectrum of [(IMes)AsP(IDipp)]PF_6 [**5**]PF_6)
6) Cyclic Voltammogram of [(IMes)AsP(IDipp)] (**4**)
7) UV/Vis spectra of **3**-**6**

C) X-ray crystallographic details

1) Single crystal X-ray details of [(IMes)AsClP(IDipp)]Cl (**3**)
2) Single crystal X-ray details of [(IMes)AsP(IDipp)] (**4**)
3) Single crystal X-ray details of [(IMes)AsP(IDipp)]PF_6 [**5**]PF_6
4) Single crystal X-ray details of dicationic compound [(IMes)AsP(IDipp)]$[GaCl_4]_2$ ([**6**]$[GaCl_4]_2$)

D) Computational details

Figure S25. Spin density distribution of the radical cation in [**5**]PF_6

Table S1. Energies for all optimized structures

Table S2. Selected bond lengths and bond angles computed in **4**, $\mathbf{5}^{+\bullet}$ and $\mathbf{6}^{2+}$

Table S3. Energies for all optimized structures

E) References

A) General synthetic procedures and analytical methods

All manipulations were performed under strictly dry argon atmosphere using standard Schlenk line techniques and dry argon- filled glove boxes. Solvents used were dried using the MBraun solvent purification system. ^{1}H, ^{13}C and ^{31}P NMR spectra were measured on Bruker AV II 300 (300 MHz), AV III 400 (400 MHz) AV III HD 500 (500 MHz). The chemical shifts are given in parts per million (δ; ppm) relative to residual solvent peaks (δ; 7.15 (C_6D_6), 5.34 (CD_2Cl_2), 3.58 and 1.73 (THF-d_8) ppm). Coupling constants (*J*) are reported in Hertz (Hz), and splitting patterns indicated as s (*singlet*), d (*doublet*), brd (broad doublet), t (*triplet*), m (*multiplet*), sept (*septet*) and br (*broad*). All spectra were measured at room temperature unless otherwise stated. Elemental analyses were carried out on a Elementar Vario Micro Cube System. Arsenic trichloride ($AsCl_3$) and Gallium trichloride ($GaCl_3$) were purchased from the Acros Organics and the other starting precursors (IDipp)$PSiMe_3$[1], (IMes)$AsCl_3$[2] were prepared according to the literature reported procedures. The X-band EPR spectra were recorded on a Bruker EMX spectrometer with an OXFORD ESR900 continuous flow cryostat. The probes were transferred into a 4 mm diameter quartz EPR tube (Wilmad 707-SQ-250M) and the spectra were simulated with EasySpin 5.1.12. Electron impact mass spectra were measured on a Thermofinnigan MAT95XL double focusing sector field mass spectrometer, ESI mass spectra on a ThermoQuest Finnigan LCQ Deca spectrometer. High-resolution electrospray (HR-MS) measurements were performed on ThermoFisher Scientific LTQ-Orbitrap Velos by direct infusion mode using a custom-made microspray-device mounted on a Proxeon nanospray ion source.

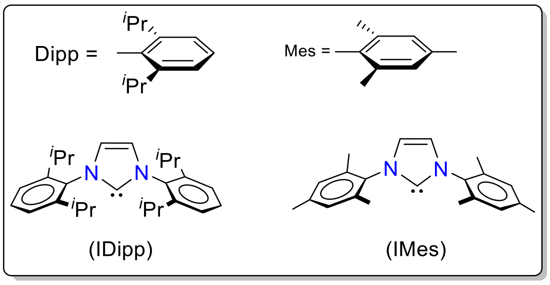

1) Preparation of (IMes)$AsCl_3$ (2)

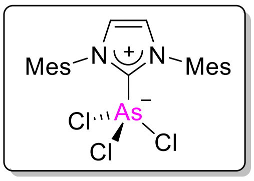

Arsenic trichloride ($AsCl_3$) (0.240 mg, 0.00 mmol) was slowly added to a stirred slurry of the carbene IMes (0.404 mg, 0.00 mmol) in *n*-hexane (50 mL) at room temperature. The resulting reaction mixture was stirred for 42h, and filtered. The precipitate obtained was then washed with *n*-hexane (2 x 10 mL) and dried under vacuum to afford an off-white solid. Yield: 0.489 g (76%). ^{1}H NMR (300.1 MHz, THF-d_8): δ = 7.59 (s, 2H, C_2N_2H), 7.04–7.96 (m, 4H, Ar-*H*), 2.36 (s, 12H, *o*-CH_3), 2.28 (s, 6H, *p*-CH_3) ppm. ^{13}C NMR (75.5 MHz, THF-d_8): δ = 156.2 (As–*C*), 141.9 (N*C*(Mes), 137.20 (*o*-C(Mes)), 135.3 (*p*-*C*(Mes)), 130.5 ($N_2C_2H_2$), 130.2 (*p*-*C*(Mes)), 21.2 (*p*-(CH_3)) and 19.5 (*o*-(CH_3) ppm. Anal. Calcd (%) for $C_{21}H_{24}AsCl_3N_2$ (485.709 g/mol): C 51.93, H 4.98 and N 5.77; Found (%): C 52.21, H 5.00 and N 5.02.

2) Preparation of [(IMes)As(Cl)P(IDipp)]Cl (3)

The carbene-phosphinidene adduct (IDipp)$PSiMe_3$ (0.306 g, 0.6177 mmol) in toluene (10 mL) was added slowly to the stirred slurry of (IMes)$AsCl_3$ (**2**) (0.300 g, 0.617 mmol) in toluene (10 mL). The resulting reaction mixture immediately turned to dark green then to brown orange with formation of an orange precipitate. The resulting reaction mixture was stirred for one day and filtered the precipitate. This solid was then washed with *n*-hexane (3 x 5 mL), dried under vacuum to afford the title compound as orange solid. Yield: 0.450 g (79%). ^{1}H NMR (CD_2Cl_2, 500.3 MHz): δ = 7.81 (s, IDipp), 7.58 (br, $N_2C_2H_2$, Dipp), 7.55-7.51-7.48 (m, 2H, IDipp), 7.23 (d, J_{HH} = 7.8 Hz, IDipp), 7.16 (br, 2H, IMes), 7.10 (br, 2H, IMes), 6.88 (br, 2H, $C_2N_2H_2$, IMes), 2.38-2.33 (br, 4H, C*H*(CH_3)$_2$), 2.31 (s, 6H, Mes-C*H*$_3$), 1.88 (br, 12H, Mes-C*H*$_3$), 1.27-1.05 (br, 24H, IDipp) ppm and shifts correspond to other products; 7.81 (s, $C_2N_2H_2$), 7,78 (d, ArH), 7.56- 7.52 (m, ArH), 7.46 (ArH), 7.39, 7.38, 7.32 (ArH), 7.31 (ArH), 6.78 (br, $C_2N_2H_2$), 2.55 (sept, J_{HH} = 6.5 Hz, C*H*(CH_3)$_2$), 2.55 (sept, J_{HH} = 6.5 Hz, C*H*(CH_3)$_2$), 2.46-2.41 (m, C*H*(CH_3)$_2$), 2.38 (s, Mes-CH_3), 2.21 (s, Mes-CH_3), 1.33 (d, IDipp), 1.28 (d, J_{HH} = 6.9, CH(C*H*$_3$)$_2$), 1.27 (d, J_{HH} = 6.7, CH(C*H*$_3$)$_2$) ppm. ^{13}C NMR (CD_2Cl_2, 125.8 MHz): δ = 166.6 (d, J_{PC} = 121.0 Hz, P–C_{NHC}), 153.0 (d, J_{PC} = 48.0 Hz, P–C_{NHC}), 152.3 (br, J_{PC} = 45.0 Hz, P-C_{NHC}), 151.7 (d, J_{PC} = 23.0 Hz, C_{NHC}), 146.3 (N*C*), 146.0 (br, N*C*), 146.3 (br, N*C*), 146.1 (br, N*C*), 145.6 (N*C*), 145.5 (N*C*), 141.7, 141,6, 140.9, 138.2, 134.7, 132.9, 132.2 (d, J_{PC} = 19.2 Hz, *C*$_2N_2H_2$), 131.7, 131.2, 130.6, 130.0, 130.1, 129.3, 128.7, 128.5, 126.7, 126.1, 125.5, 125.5, 125.1, 124.9, 124.8, 124.5, 29.7 (*C*H(CH_3)$_2$), 29.5 (*C*H(CH_3)$_2$), 29.3 (*C*H(CH_3)$_2$), 26.7 (*C*H$_3$), 25.3 (*C*H$_3$), 24.8 (*C*H$_3$), 23.6 (*C*H$_3$), 22.6 (*C*H$_3$), 21.4, 21.3, 21.2, 18.2 (br, *o*-CH_3), 17.8 (br, *p*-CH_3) ppm. ^{31}P NMR (CD_2Cl_2, 202.5 MHz); δ = 132.1 (**3**, (IDipp)*P*Cl)), 20.25 (**3',** (IDipp)*P*ClAs), 16.54 (**3'',** *P*AsCl_2), 1.80 (**3''',** (IDipp)*P*) ppm. UV/Vis (THF, 298K, λ(nm) ε($M^{-1}cm^{-1}$)): 451 (2841.3). Anal. Calcd (%) for $C_{48}H_{61}AsPN_4Cl_2$ (MW: 870.8260 g/mol); C 66.20, H 7.06 and N 6.43; Found (%): C 66.05, H 7.09 and N 5.69.

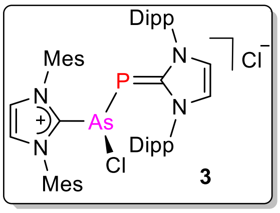

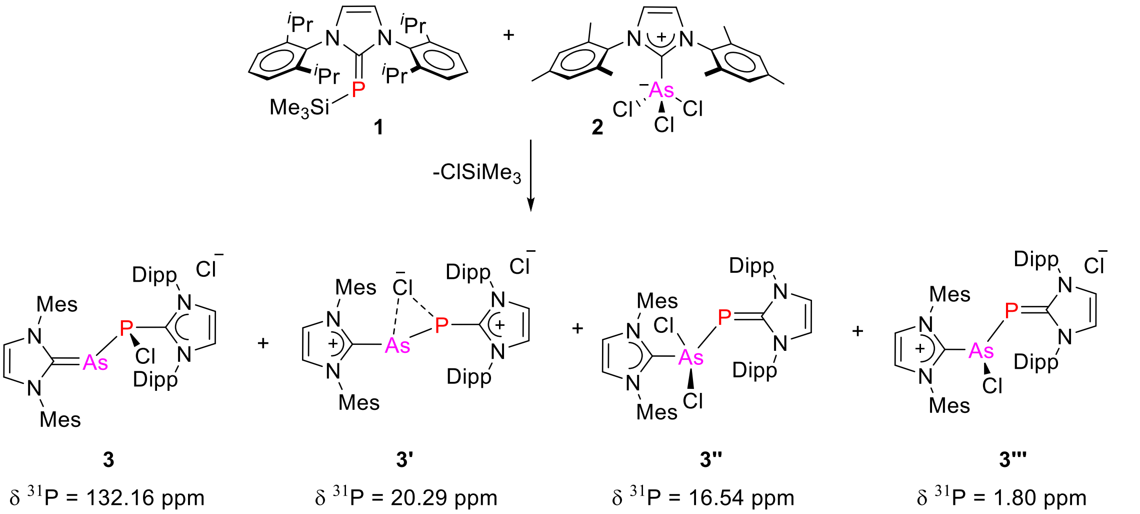

Scheme S1: Likely species of **3** at room temperature in CD_2Cl_2 and their ^{31}P NMR chemical shifts.

3) Preparation of [(IMes)AsP(IDipp)] (4)

Freshly distilled dry THF (15 mL) was added to a Schlenk tube containing [(IMes)As(Cl)P(IDipp)]Cl (0.150 g, 0.172 mmol) and potassium graphite (0.047 g, 0.344 mmol). After stirring the resulting dark green-black slurry for 6h at room temperature, solvent was removed under reduced pressure and the

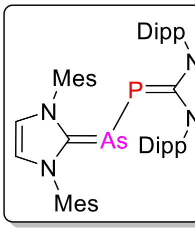

4

residue obtained was extracted with *n*-hexanes (3 x 5 mL) and concentrated to 4 mL. After storage at –32º C for three days, crystalline solid was settled; it was then filtered and quickly washed with cold *n*-hexane to obtain the title compound as dark red solid. Yield; 0.051 g (37%). ^{1}H NMR (500 MHz, C_6D_6): δ = 7.32-7.17 (m, 2H, *p-H*(Dipp)), 7.01 (d, J_{HH} = 7.3 Hz, *m-H*(Dipp)), 6.67 (br, 4H, *m-H*(IMes), 6.08-5.91 (br, 2H, $N_2C_2H_2$, IDipp), 5.83 (br, 2H, $N_2C_2H_2$, IMes), 3.18 (sept, 4H, J_{HH} = 6.8 Hz, $CH(CH_3)_2$), 2.19 (s, 6H, *p*-CH_3, IMes), 2.15 (s, 12H, *p*-CH_3, IMes), 1.46 (d, 12H, J_{HH} = 6.7 Hz, $CH(CH_3)_2$) and 1.19 (d, 12H, J_{HH} = 6.7 Hz, $CH(CH_3)_2$) ppm. ^{13}C NMR (125.8 MHz, C_6D_6): δ = 148.5 (NC(Dipp), 146.3 (NC(IMes), 137.8 (*o*-C(Dipp), 136.9 (N*C*(Mes), 135.0 (*o-C*(Mes)), 129.1 (*m-C*(Mes)), 128.9 (*p-C*(Mes), 123.7 (*p-C*(Dipp)), 123.5 ((*m-C*(Dipp)),121.5 ($C_2N_2H_2$), 118.8 ($C_2N_2H_2$), 28.9 ($CH(CH)_3$), 24.9 ($CH(CH)_3$), 23.6 ($CH(CH)_3$), 21.5 (*p*-$CH)_3$) and 18.6 (*o*-$CH)_3$) ppm. ^{31}P NMR (121.5 MHz, C_6D_6): δ = –60.63 ppm. UV/Vis (THF): λ_{max} (ε) = 431 nm (3409.0 M^{-1} cm^{-1}). Anal. Calcd (%) for $C_{48}H_{60}N_4PAs$ (MW: 798.9122 g/mol): C 72.16, H 7.57 and N 7.01; Found (%): C 72.21, H 7.97 and N 6.62.

4) Preparation of [(IMes)AsP(IDipp)]PF_6 [5]PF_6

A Schlenk tube containing [(IMes)AsP(IDipp)] (**4**) (0.040 g, 0.050 mmol) in THF (10 mL) was added ferrocenium hexfluorophosphate [Fc]PF_6 (0.016 g, 0.050 mmol) dissolved in THF (5 mL). The color of the reaction mixture turned to dark green immediately. The resulting reaction mixture was stirred for 5 h

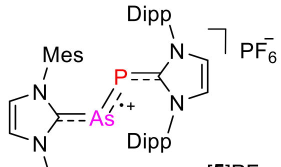

[**5**]PF_6

at room temperature and filtered. The solvent was removed from the filtrate under vacuum to give a dark green solid. This solid was further washed with toluene (1 mL x 4) followed by *n*-hexane until the washings were colorless. The dark residue was dried under vacuum to afford the title compound. Yield: 0.018 g (38%). Dark green crystals of [**5**]PF_6 were obtained from the solvent mixture of hexane layer over dichloromethane solution. EPR data (CH_2Cl_2, 298 K, 2.00865 GHz): g_{iso} = 2.0246. Hyperfine couplings; A^{75}As = 21.7 G (60.7 MHz or 2.17 mT), A^{31}P = 42.2 G (118.2 MHz (or) 4.22 mT), and A^{14}N =1.5 G (4.1 MHz (or) 0.147 mT). UV/Vis (THF): λ_{max} (ε) = 457 (2890.0), 622 nm (2650.0 M^{-1} cm^{-1}). Anal. Calcd (%) for $C_{48}H_{60}F_6N_4P_2As$ (943.90 g/mol): C 61.08, H 6.41, 5.94; Found (%): C 61.20, H 6.46 and N 5.57.

5) Preparation of [(IMes)AsP(IDipp)][$GaCl_4$]$_2$ [6][$GaCl_4$]$_2$

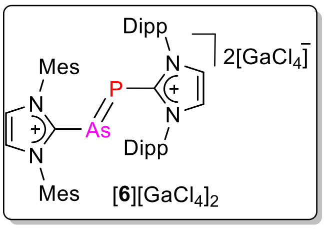

[**6**][$GaCl_4$]$_2$

Gallium trichloride ($GaCl_3$) (0.030 g, 0.172 mmol) dissolved in THF (5 mL) was slowly added to a stirred solution of [(IMes)As(Cl)P(IDipp))]Cl (**3**) (0.075 g, 0.086 mmol) dissolved in THF (10 mL) at room temperature. The resulting reaction mixture was stirred for one day at room temperature and filtered. The solvent was removed from the filtrate to get a red oily residue which was quickly washed with fluorobenzene (2 x 2 mL) followed by

diethyl ether and *n*-hexane (2 x 3 mL). The remaining residue was then dried under vacuum to get the title compound as dark orange solid. Yield: 0.049 g (47%). ^{1}H NMR (300 MHz, CD_2Cl_2): δ = 8.28 (s, 2H, N_2C_2-*H*, IDipp), 7.89 (s, 2H, N_2C_2-*H*, IMes), 7.69 (t, 2H, J_{HH} = 7.9 Hz, Ar-*H*, IDipp), 7.38 (d, 4H, J_{HH} = 7.5 Hz, Ar-*H*, IDipp), 6.99 (br, 4H, Ar-*H*, IMes), 2.36 (s, 6H, *p*-C*H*$_3$), 2.10 (broad sept, 4H, J_{HH} = 6.6 Hz, C*H*(CH)$_3$, IDipp), 1.87 (s, 12H, *o*-C*H*$_3$, IMes), 1.22 (d, 12H, J_{HH} = 7.0 Hz, CH(C*H*)$_3$, IDipp) and 0.82 (d, 12H, J_{HH} = 6.6 Hz, CH(C*H*)$_3$, IDipp) ppm. ^{13}C NMR (125.8 MHz, CD_2Cl_2): δ = 152.2 (brd, $^{1}J_{PC}$ = 100.1 Hz, P–C_{NHC}), 151.3 (brd, $^{2}J_{PC}$ = 21 Hz, As–C_{NHC}), 145.7 (N*C*(Dipp)), 144.0 (N*C*(Mes)), 134.6 (*o*-*C*(Dipp)), 134.4 (*o*-*C*(Mes)), 131.3 (*m*-*C*(Mes)), 131.1, 130.4 ($N_2C_2H_2$), 130.0 ($N_2C_2H_2$), 129.7 (*o*-*C*(Dipp), 126.5 (*m*-*C*(Dipp)), 29.7 (*C*H(CH)$_3$), 25.1 (CH(*C*H)$_3$), 23.6 (CH(*C*H)$_3$), 21.5 (*p*-*C*H$_3$) and 17.9 (*o*-*C*H$_3$) ppm. ^{31}P NMR (121.5 MHz, CD_2Cl_2): δ = 475.5 ppm. UV/Vis (THF): λ_{max} (ε) = 443 nm (4127.1 M^{-1} cm^{-1}). Anal. Calcd (%) for $C_{48}H_{61}AsCl_8Ga_2N_4P$ (MW: 1222.99 g/mol): C 47.14, H 5.03 and N 4.58; Found (%): C 46.63, H 5.08 and N 3.77.

B) NMR and other spectra of compounds 2-6

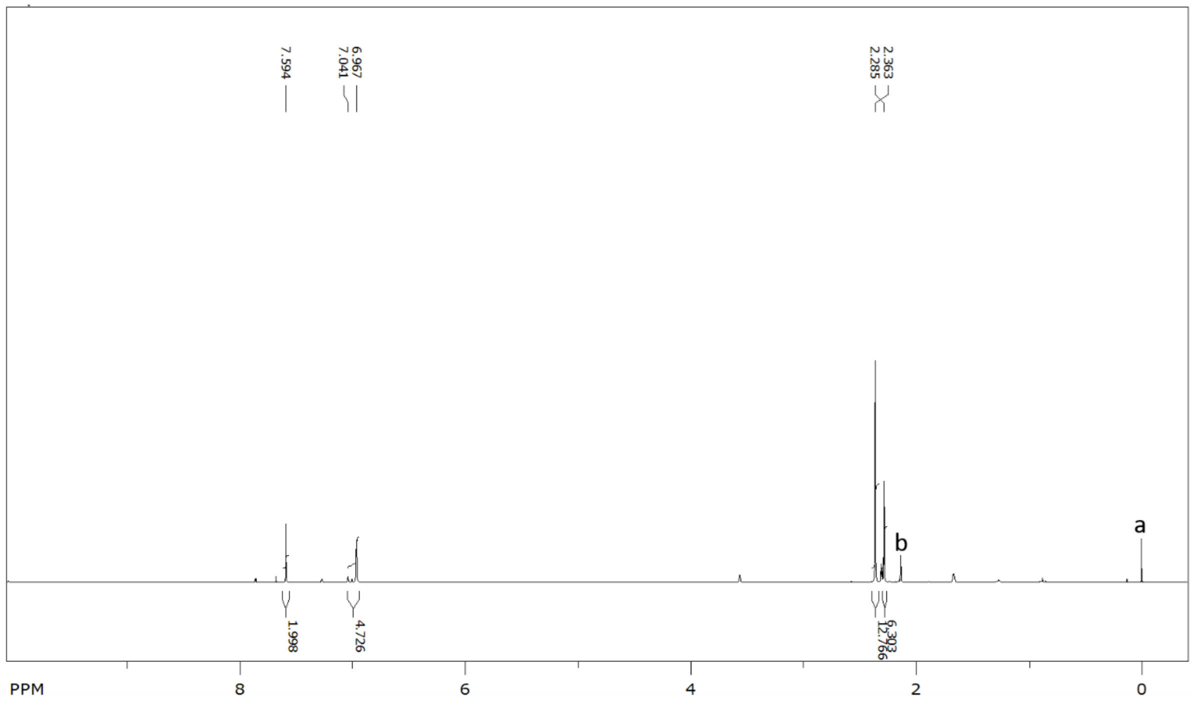

Figure S1. ^{1}H NMR spectrum of (IMes)$AsCl_3$ (**2**) in THF-d_8 at room temperature (other prominent signals. a =TMS, b = unknown impurity).

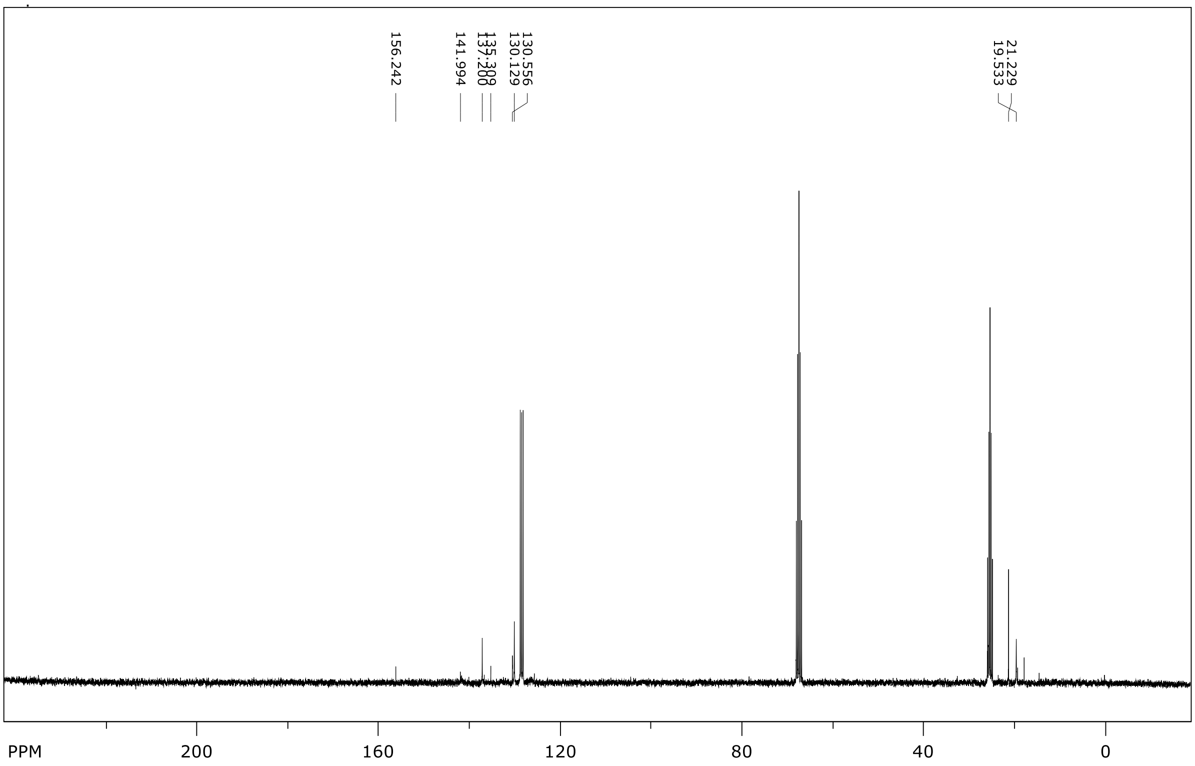

Figure S2. ^{13}C NMR spectrum of the adduct (IMes)$AsCl_3$ (**2**) in THF-d_8 at room temperature.

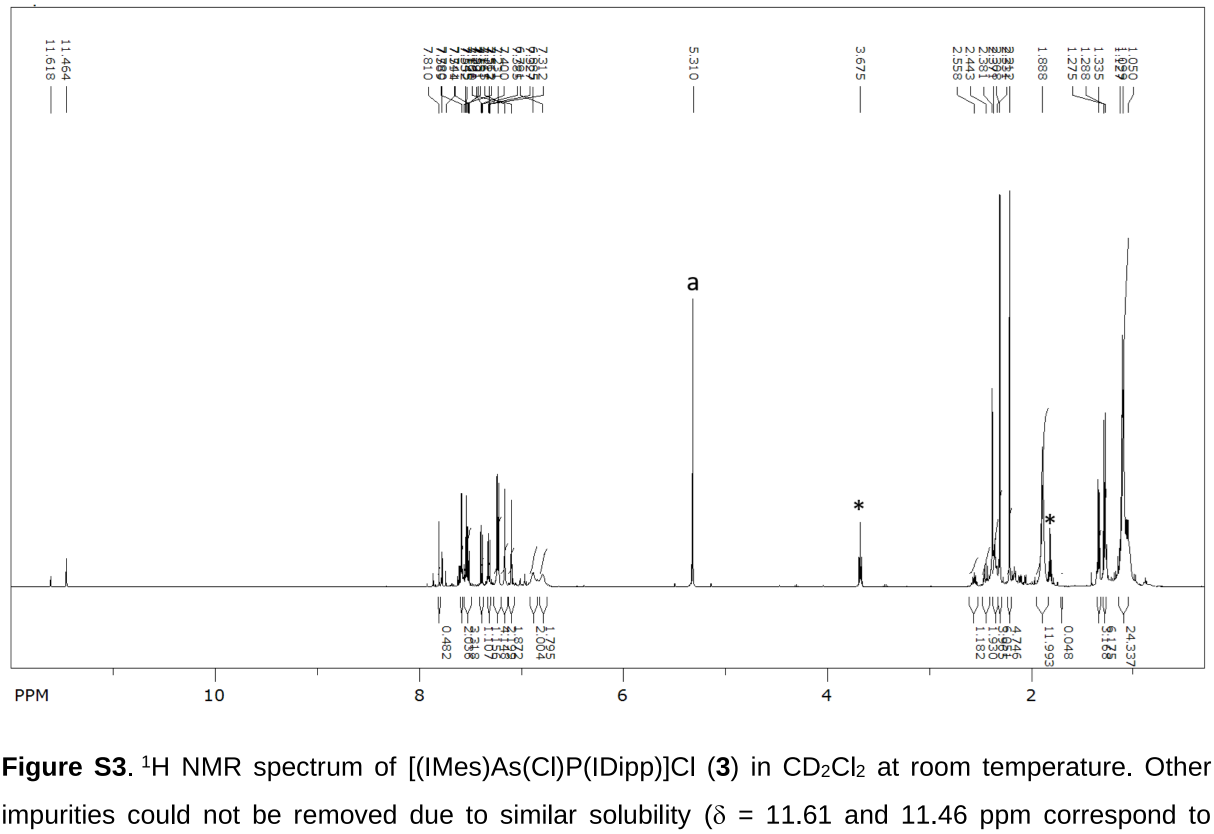

Figure S3. ^{1}H NMR spectrum of [(IMes)As(Cl)P(IDipp)]Cl (**3**) in CD_2Cl_2 at room temperature. Other impurities could not be removed due to similar solubility (δ = 11.61 and 11.46 ppm correspond to imidazolium protons, a = CH_2Cl_2, * = THF).

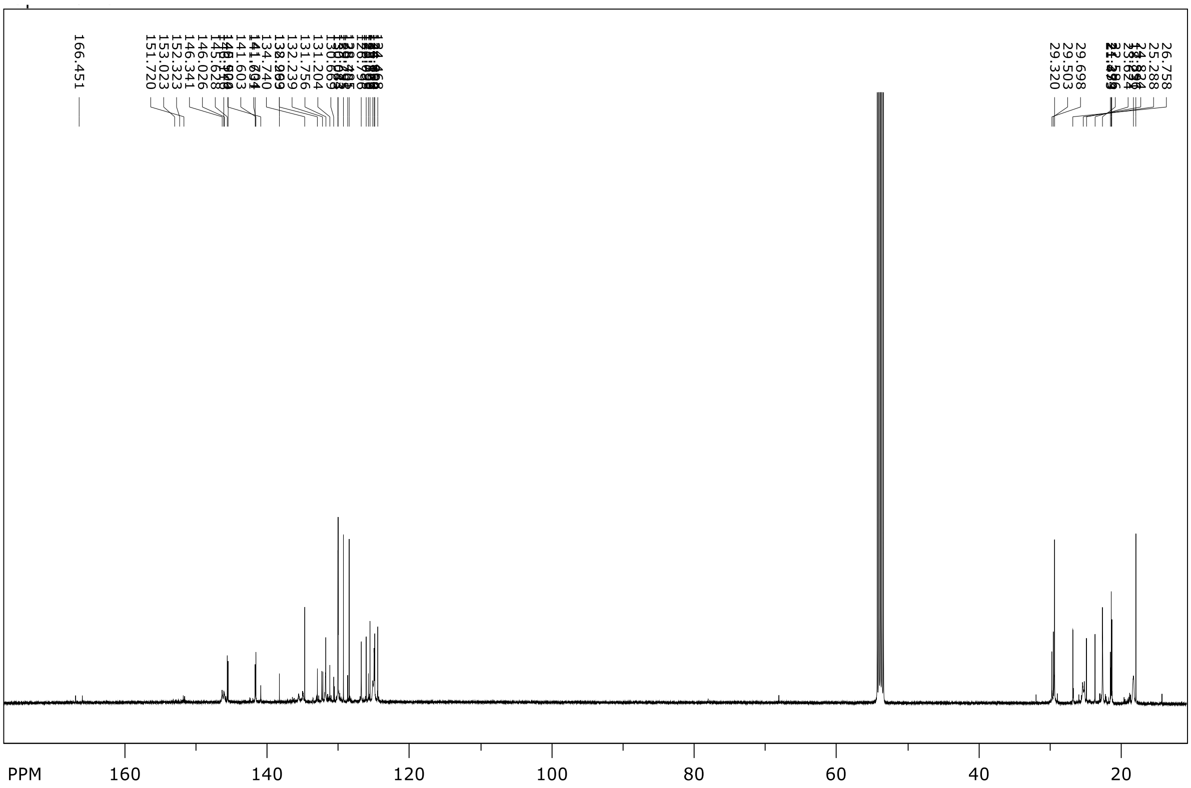

Figure S4. ^{13}C NMR spectrum of [(IMes)As(Cl)P(IDipp)]Cl (**3**) in CD_2Cl_2 at room temperature.

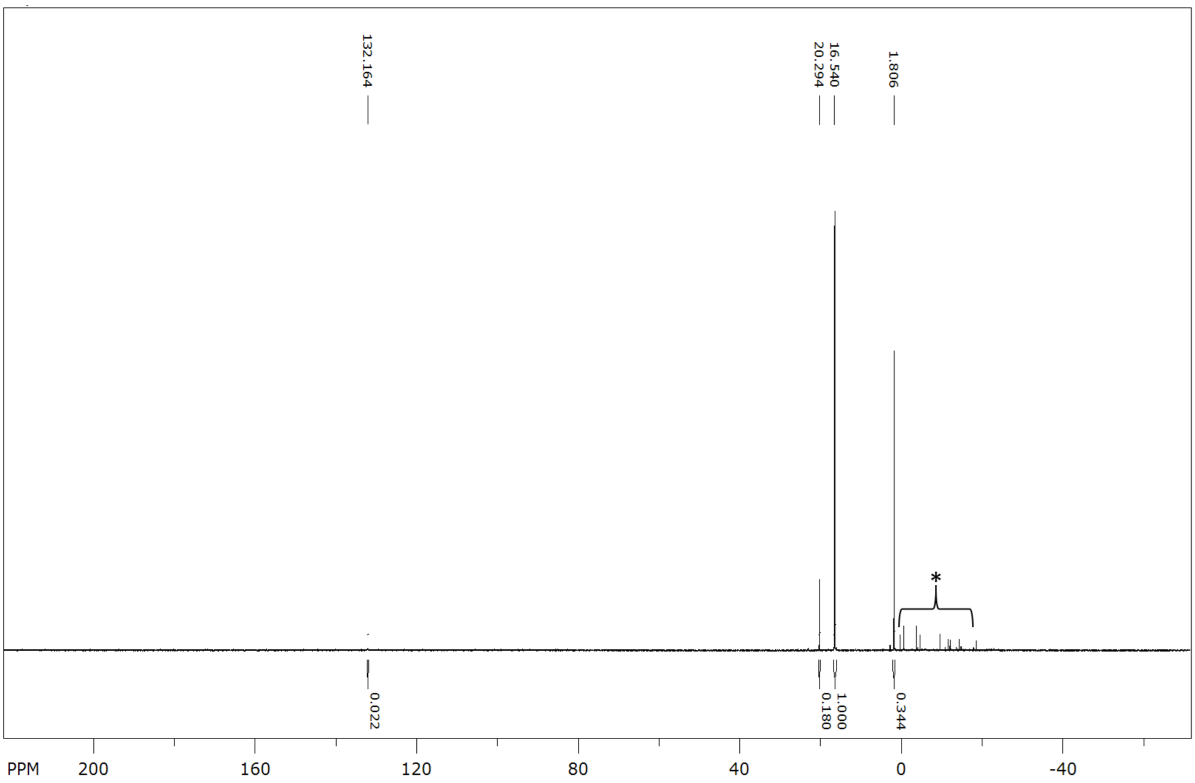

Figure S5. ^{31}P NMR spectrum of [(IMes)As(Cl)P(IDipp)]Cl (**3**) in CD_2Cl_2 at room temperature (* = other impurities could not be removed due to similar solubility).

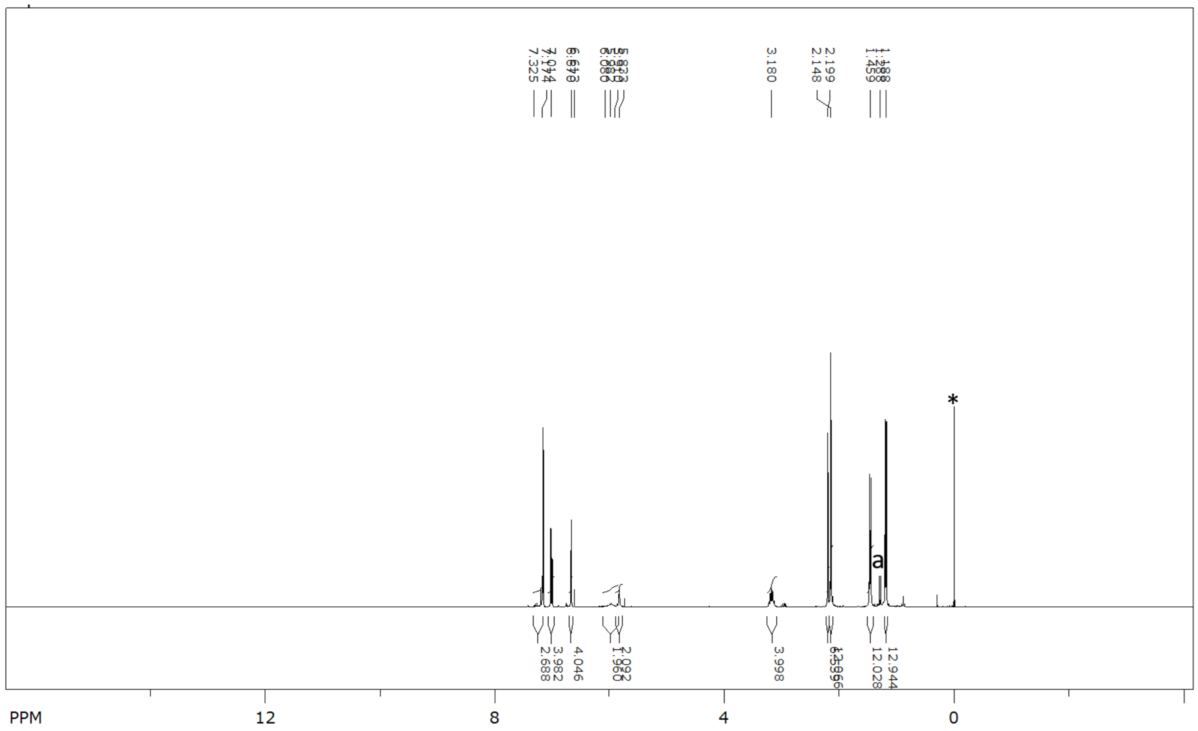

Figure S6. ^{1}H NMR spectrum of [(IMes)AsP(IDipp)] (**4**) in C_6D_6 at room temperature (* = TMS, a = free carbene, IDipp).

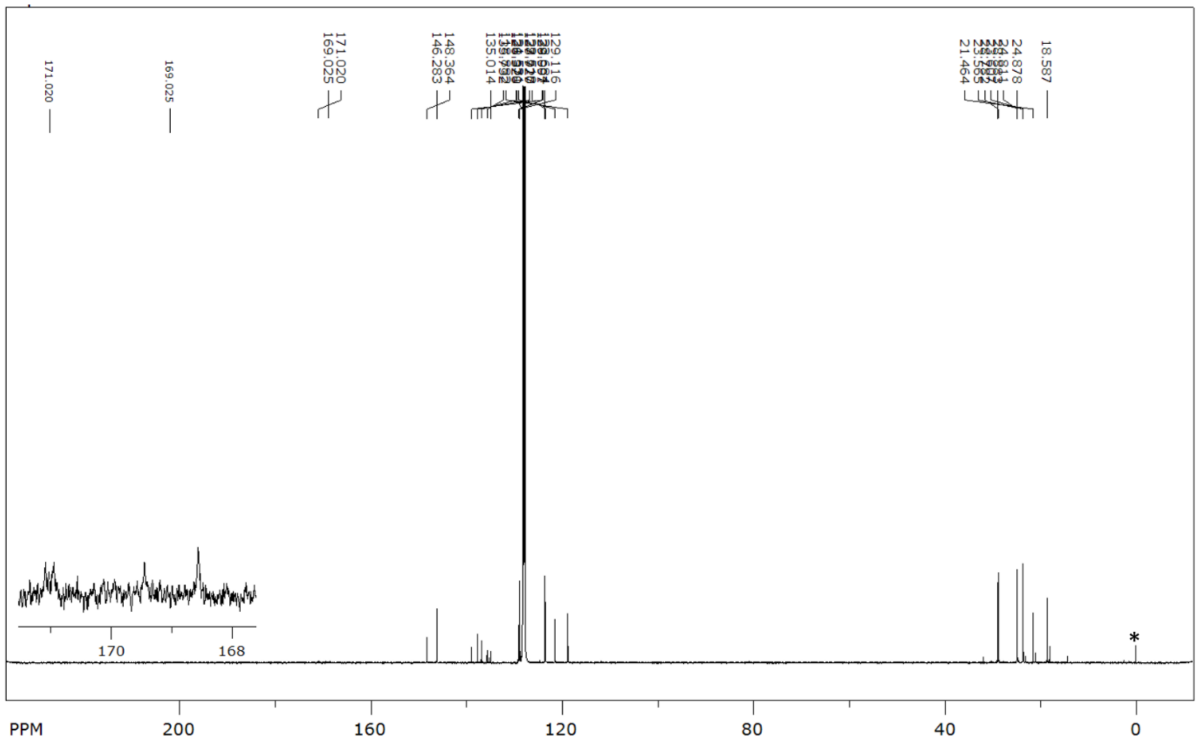

Figure S7. ^{13}C NMR spectrum of [(IMes)AsP(IDipp)] (**4**) in C_6D_6 at room temperature (* = TMS).

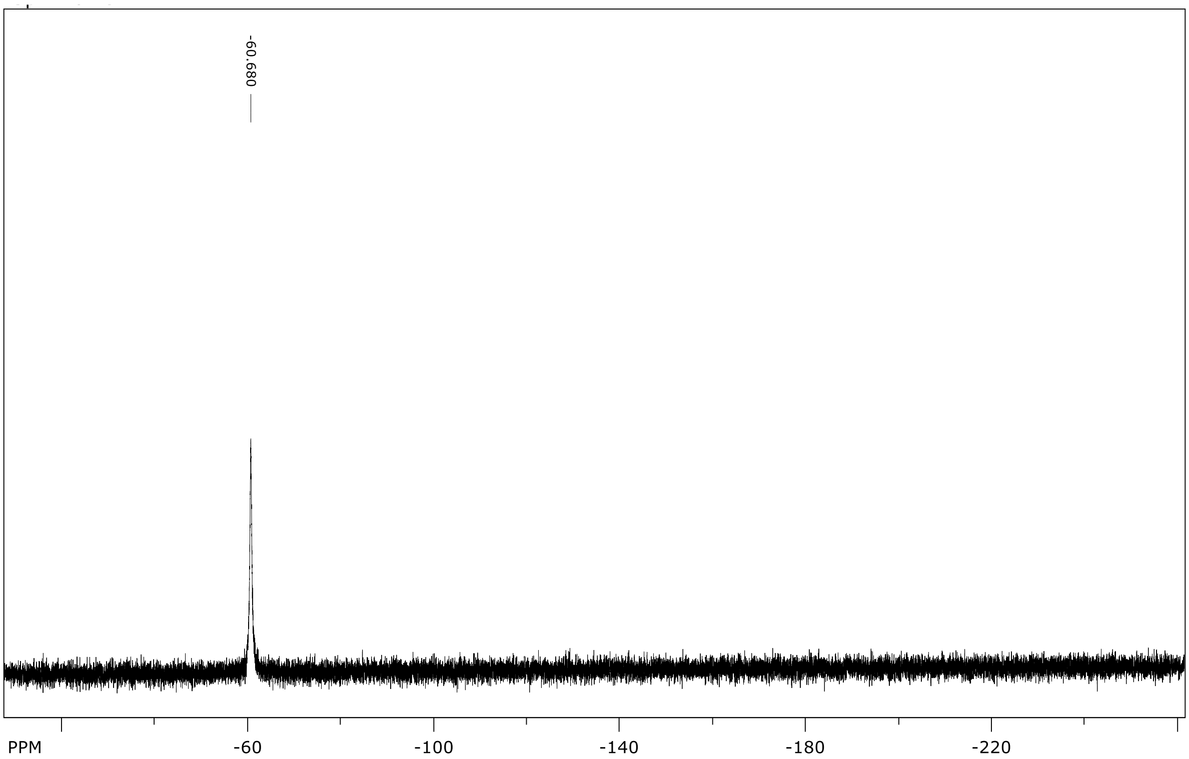

Figure S8. ^{31}P NMR spectrum of [(IMes)AsP(IDipp)] (**4**) in C_6D_6 at room temperature.

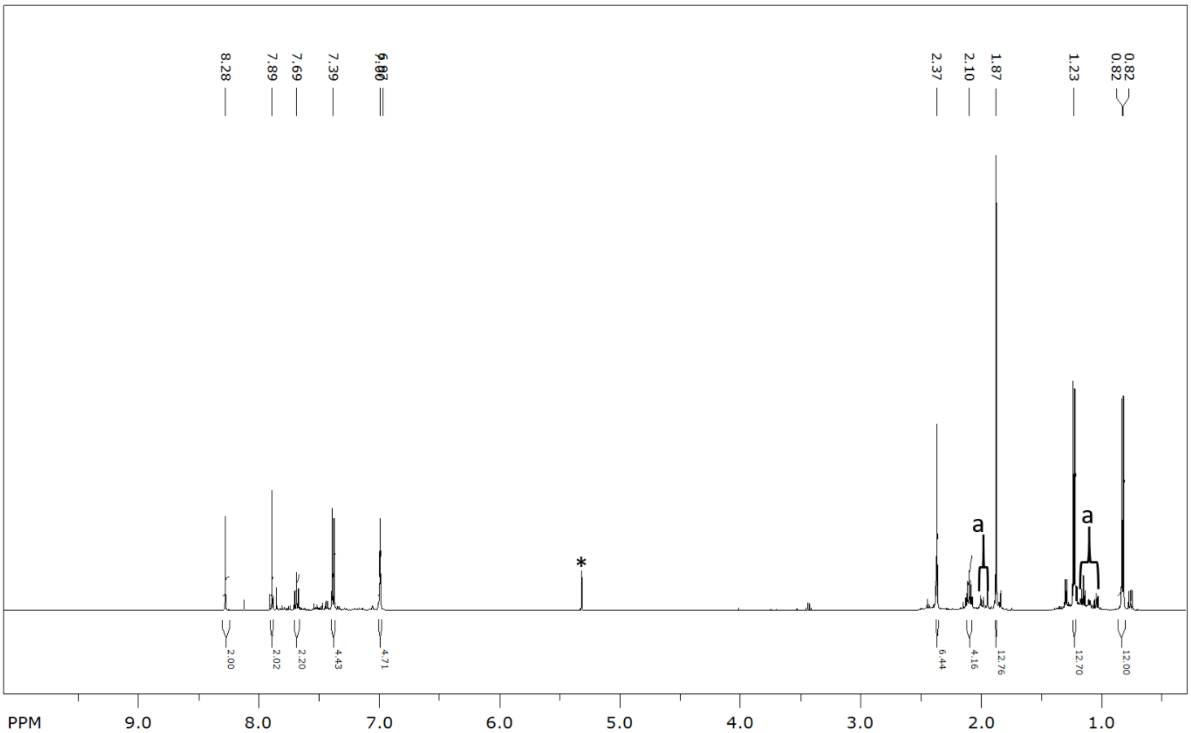

Figure S9. ^{1}H NMR spectrum of [(IMes)As=P(IDipp)][$GaCl_4$]$_2$ ([**6**][$GaCl_4$]$_2$) in CD_2Cl_2 at room temperature (a = impurity, * = CH_2Cl_2).

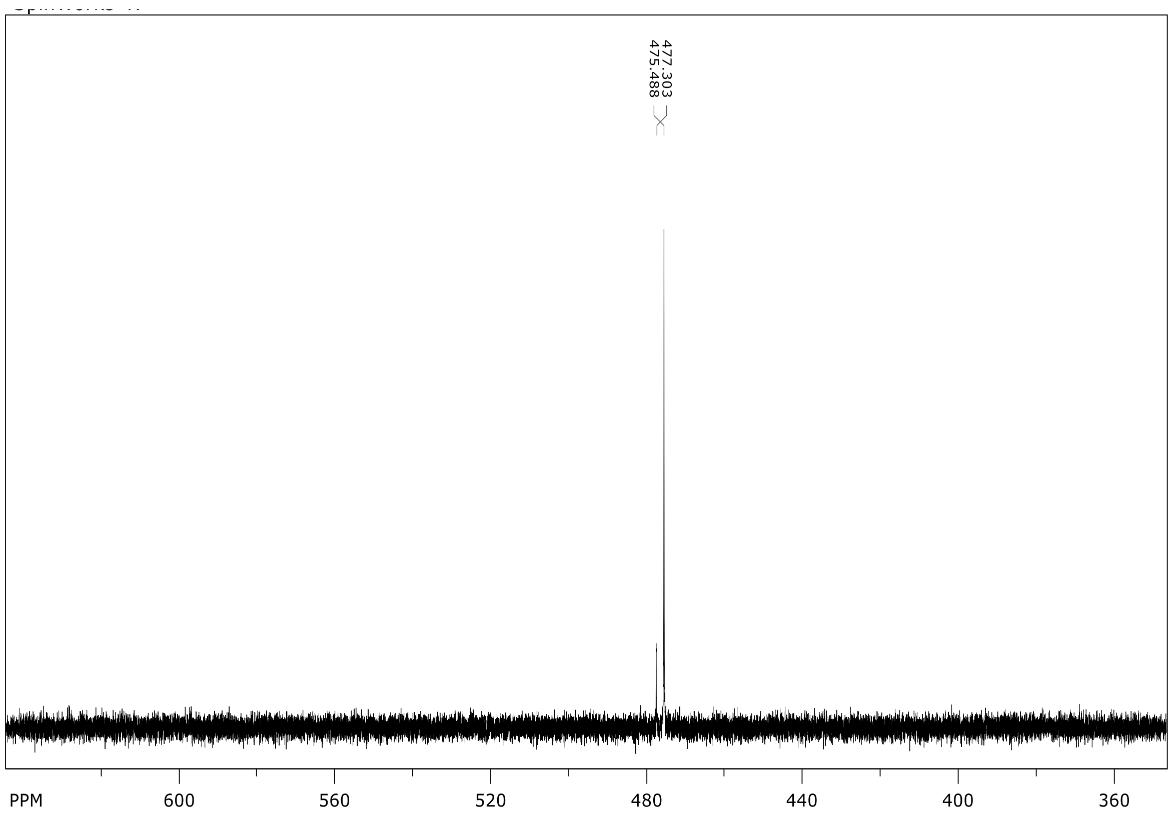

Figure S10. $^{31}P\{^{1}H\}$ NMR spectrum of $[(IMes)As{=}P(IDipp)][GaCl_4]_2$ ([**6**]$[GaCl_4]_2$) in CD_2Cl_2 at room temperature (impurity at 477.3 ppm could not be removed despite several purification attempts).

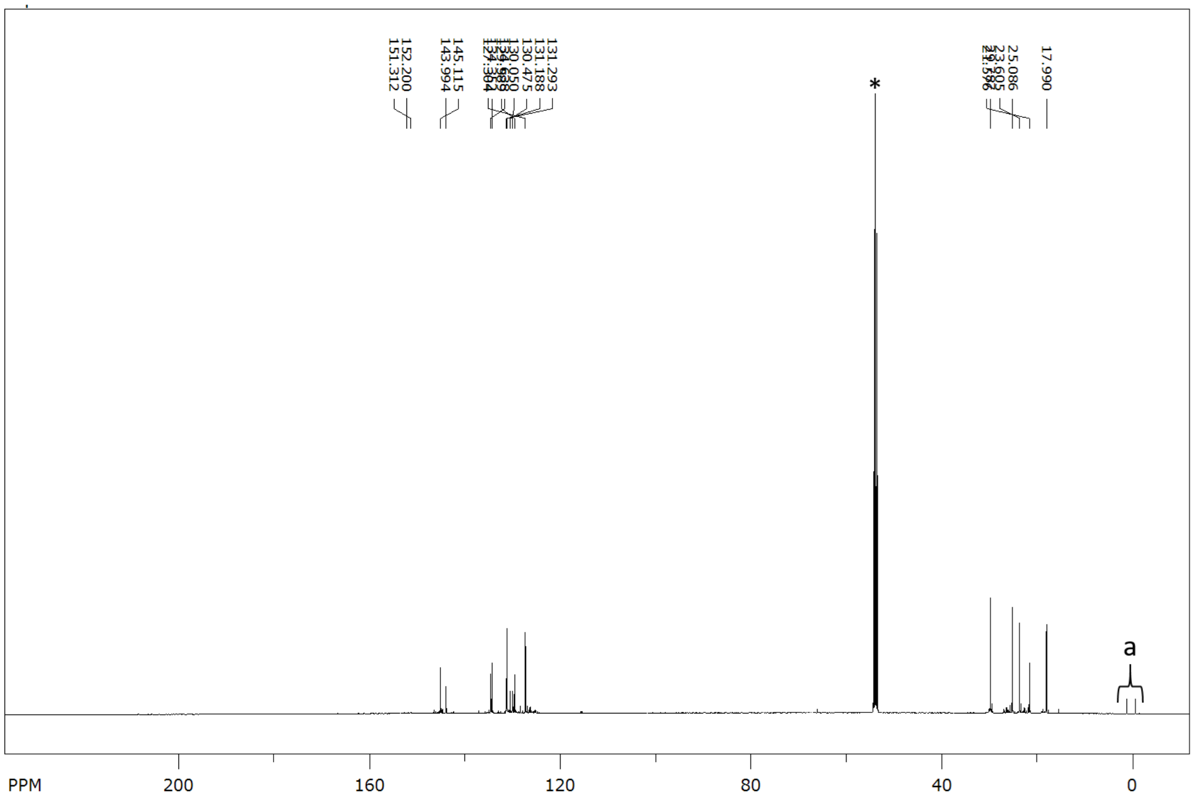

Figure S11. ^{13}C NMR spectrum of $[(IMes)As{=}P(IDipp)][GaCl_4]_2$ ([**6**]$[GaCl_4]_2$) in CD_2Cl_2 at room temperature (* = residual solvent, a = impurity).

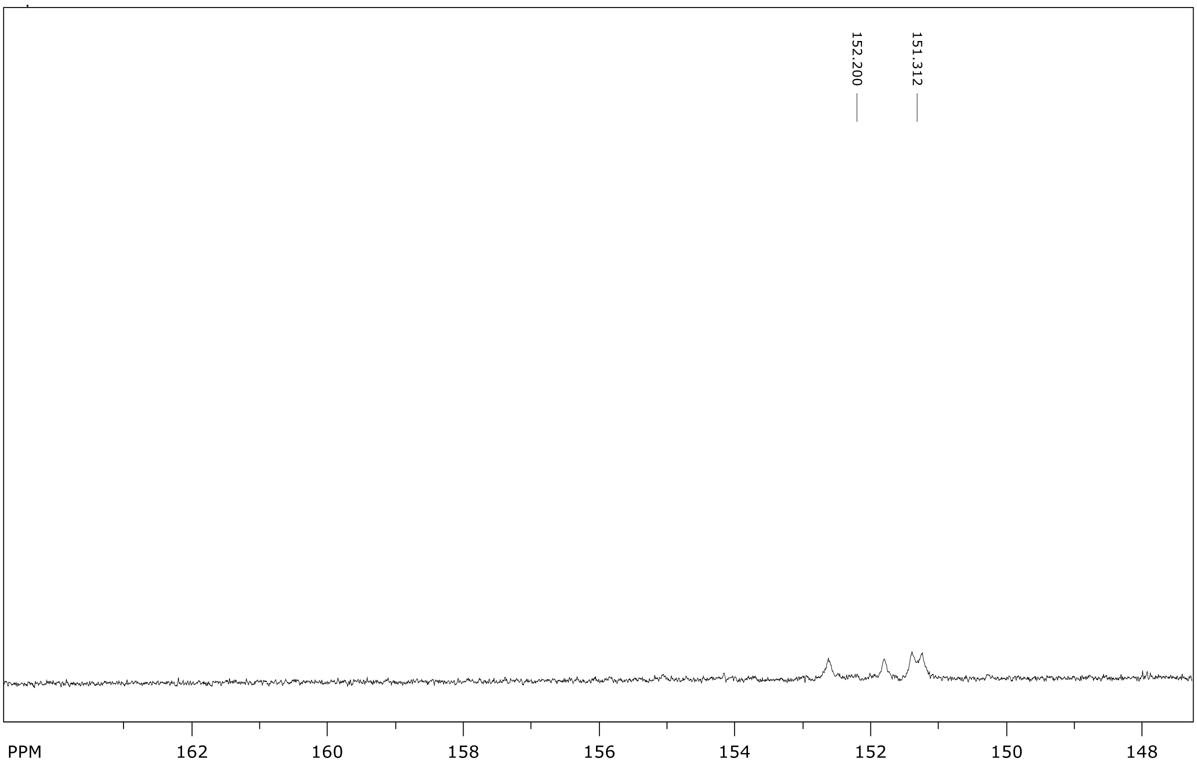

Figure S12. ^{13}C (expanded) NMR spectrum of [(IMes)As=P(IDipp)][$GaCl_4$]$_2$ ([**6**][$GaCl_4$]$_2$) in CD_2Cl_2 at room temperature.

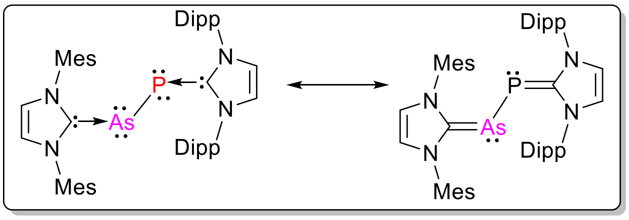

Chart S1: Canonical forms of [(IMes)AsP(IDipp)] (**4**).

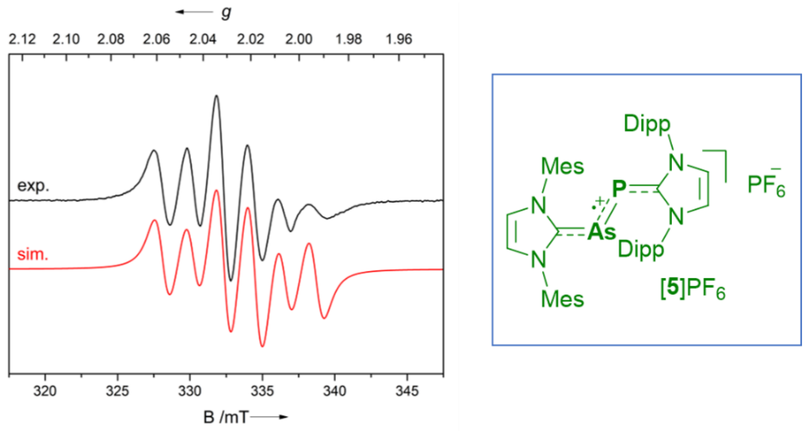

Figure S13. Experimental (black) and simulated (red) EPR spectrum (left) of [(IMes)AsP(IDipp)]PF_6 [**5**]PF_6 in CH_2Cl_2 (298K, 9.448437 GHz).

Details of EPR measurement of the radical cation [**5**]PF_6

The X-band EPR spectra were recorded on a Bruker EMX spectrometer at room temperature in a dichloromethane solution. The samples were transferred into a 4 mm diameter quartz EPR tube (Wilmad 707-SQ-250M and the experiment was recorded at 9.448437 GHz, using a modulation frequency of 100 kHz, a modulation amplitude of 8G and a microwave power of 7.989 mW.) The spectra were computed with Matlab-R2016b-9.1.0.441655 using the EasySpin 5.2.23 package.[3]

```
Sys.S = 1/2;

Sys.Nucs = '31P, 75As, N';

Sys.n = [1 1 4];

g_1 = 2.0246;

Sys.g = [g_1];

A_As = 60.7387;

A_P1 = 118.189;

A_N = 4.13389;

Sys.A = [A_P1; A_As; A_N];

Koppl_As = mhz2mt (A_As);

Koppl_P = mhz2mt (A_P1);

Koppl_N = mhz2mt (A_N);

Sys.lwpp = [0 0.932964];

Exp.mwFreq = 9.448437;

Exp.Range = [317.5 347.5];

Exp.nPoints = 2048;

Exp.Harmonic = 1;

SimOpt.Verbosity = 2;
```

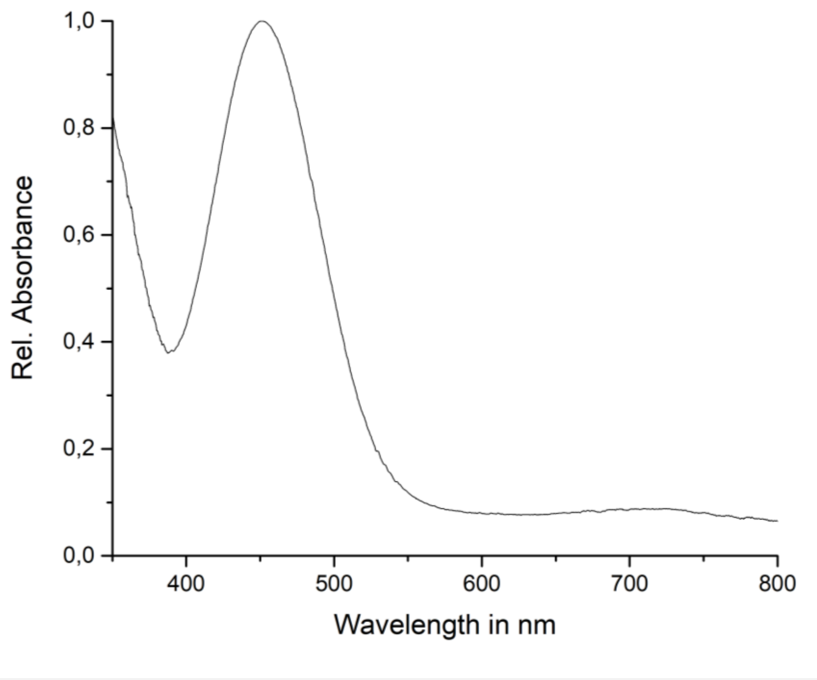

Figure S14. UV/Vis spectrum of [(IMes)As(Cl)P(IDipp)]Cl (**3**) in THF solution (0.125 M, 298K).

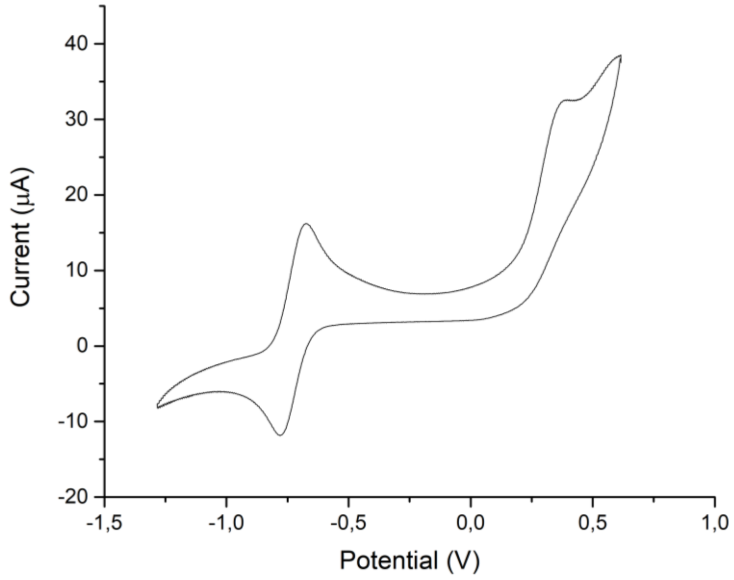

Figure S15. Cyclic voltammogram of [(IMes)AsP(IDipp)] (**4**) in THF solution (2 mM, 298K, 0.1 M *n*Bu_4PF_6, scan rate 0.1 mV/s, referenced against Fc/Fc^+. The left reversible redox couple appears at a half wave potential of $E_{1/2}$ = -0.689 mV. The right irreversible oxidation occurs at a potential of E = 0.382 mV.

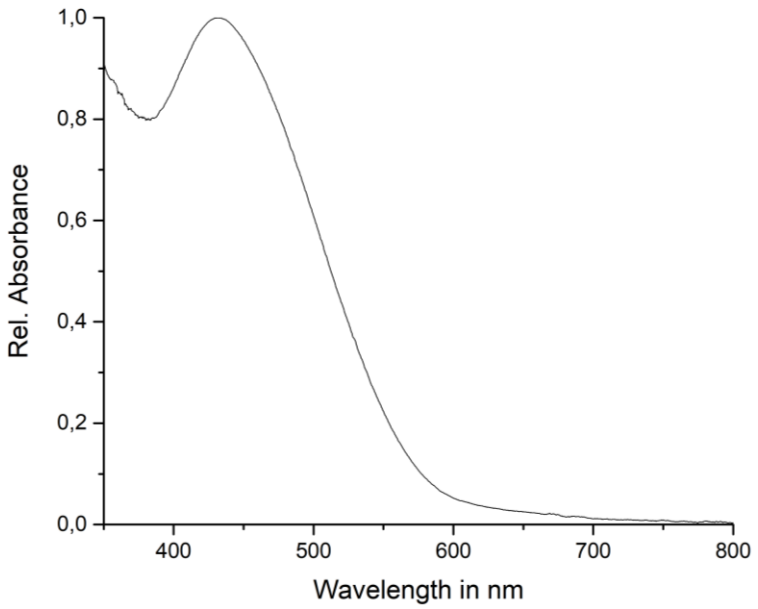

Figure S16. UV/Vis spectrum of [(IMes)AsP(IDipp)] (**4**) recorded in THF solution (0.125 M, 298K).

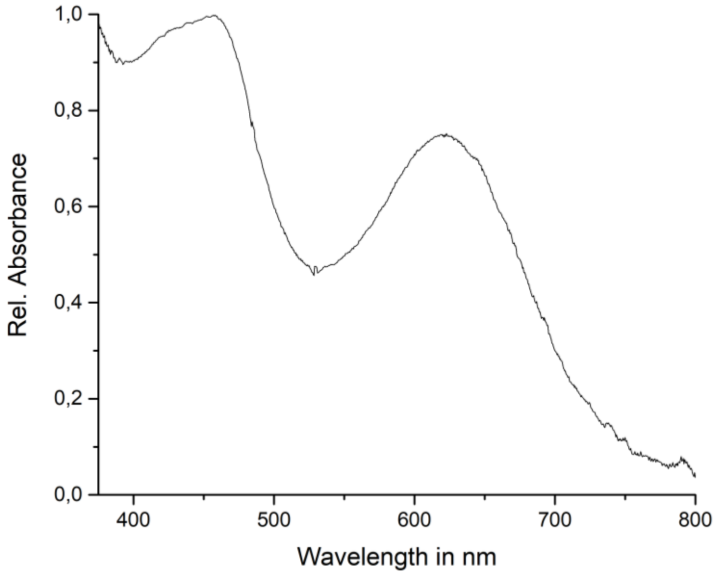

Figure S17. UV/Vis spectrum of [(IMes)AsP(IDipp)][PF_6] (**5**) recorded in THF solution (0.125 M, 298K).

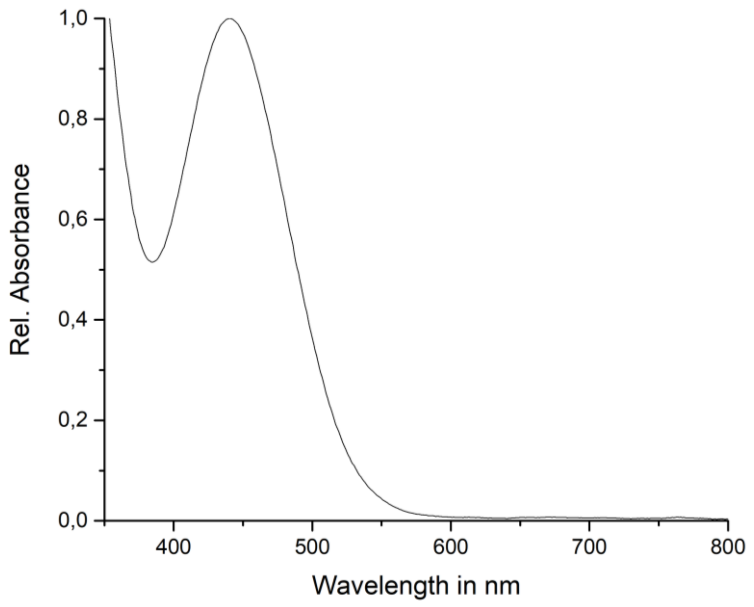

Figure S18. UV/Vis spectrum of $[(IMes)As{=}P(IDipp)][GaCl_4]_2$ (**6**) in THF solution (0.125 M, 298K).

C) X-ray crystallographic details

The crystals were mounted on top of a human hair or a glass needle (polymorph **B** of compound **3**) with per-fluorinated inert oil. Data of compound **4** and polymorph **B** of compound **3** were recorded on an Oxford Diffraction Xcalibur diffractometer equipped with a Mo-fine-focus tube, a graphite monochromator and an Eos CCD detector. All other data were recorded with mirror focused Cu-Kα radiation on an Oxford Diffraction Xcalibur diffractometer equipped with a micro-focus tube and an Atlas CCD detector. Data reduction was performed with CrysalisPro.[4] Absorption correction was based on multi-scans and except for compound **4** additionally face indexation and integration on a Gaussian grid was applied. The structure was solved by intrinsic phasing with SHELXT-2014/5[5] and refined on F^2 using the program SHELXL-2017/1[6] in WinGX v2014.1.[7] H atoms were placed in idealized positions and refined using a riding model.

Single crystal X-ray details of [(IMes)As(Cl)P(IDipp)]Cl (3): Crystal Structure of [(IMes)As(Cl)P(IDipp)]Cl·3CH_2Cl_2 (3·3CH_2Cl_2): Polymorph A

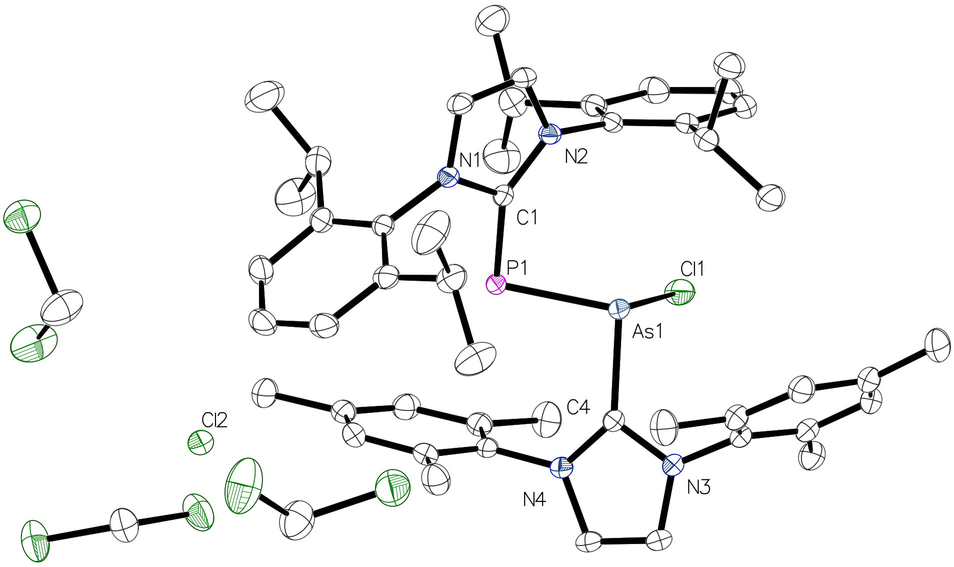

Figure S19. The asymmetric unit of polymorph A of [(IMes)As(Cl)P(IDipp)]Cl·3CH_2Cl_2 (**3**·3CH_2Cl_2). Hydrogen atoms are omitted for clarity.

CCDC Number	1940130	
Empirical formula	$C_{51}H_{66}AsCl_8N_4P$	
Formula weight	1124.56	
Temperature	100(2) K	
Wavelength	1.54184 Å	
Instrument (scan mode)	Oxford Diffraction Xcalibur, Atlas, Nova (ω scan)	
Crystal system	Triclinic	
Space group	$P\bar{1}$	
Unit cell dimensions	a = 11.2245(2) Å	α = 95.512(2)°
	b = 15.2738(4) Å	β = 92.081(2)°
	c = 16.4109(4) Å	γ = 93.093(2)°
Volume	2793.97(11) $Å^3$	
Z	2	
Density (calculated)	1.337 Mg/m^3	
Absorption coefficient	4.897 mm^{-1}	
F(000)	1168	
Crystal habitus	irregular (orange)	
Crystal size	0.396 x 0.116 x 0.015 mm^3	
Theta range for data collection	3.777 to 76.237°	
Index ranges	-14<=h<=14, -19<=k<=19, -20<=l<=20	
Reflections collected	92668	
Independent reflections	11657 [R(int) = 0.0428]	
Completeness to θ = 67.684°	100.0 %	
Absorption correction	Gaussian	
Max. and min. transmission	0.983 and 0.955	
Refinement method	Full-matrix least-squares on F^2	
Data / restraints / parameters	11657 / 0 / 600	
Goodness-of-fit on F^2	1.032	
Final R indices [I>2σ(I)]	R1 = 0.0355, wR2 = 0.0941	
R indices (all data)	R1 = 0.0381, wR2 = 0.0962	
Largest diff. peak and hole	1.360 and -0.903 e·$Å^{-3}$	
Crystallisation Details:	CH_2Cl_2/*n*-hexane at r.t.	

Single crystal X-ray details of [(IMes)As(Cl)P(IDipp)]Cl (3): Crystal Structure of [(IMes)AsClP(IDipp)]Cl·3CH_2Cl_2 (3·3CH_2Cl_2): Polymorph B

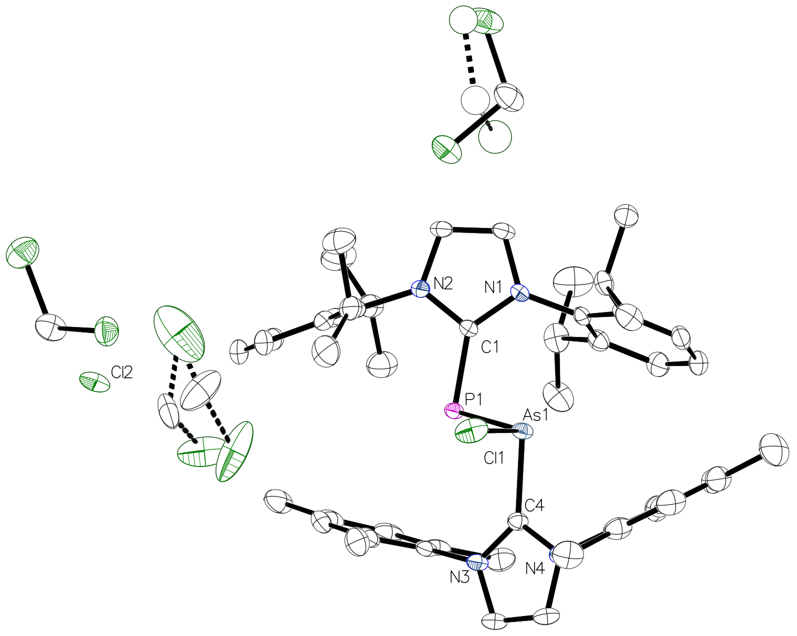

Figure S20. The asymmetric unit of polymorph B of [(IMes)As(Cl)P(IDipp)]Cl·3CH_2Cl_2 (**3**·3CH_2Cl_2). Hydrogen atoms are omitted for clarity.

CCDC Number	1940131	
Empirical formula	$C_{51}H_{66}AsCl_8N_4P$	
Formula weight	1124.56	
Temperature	100(2) K	
Wavelength	0.71073 Å	
Instrument (scan mode)	Oxford Diffraction Xcalibur, Eos (ω scan)	
Crystal system	Triclinic	
Space group	$P\bar{1}$	
Unit cell dimensions	a = 12.4079(4) Å	α = 99.043(4)°
	b = 12.7918(4) Å	β = 106.793(4)°
	c = 19.7886(8) Å	γ = 106.245(2)°
Volume	2788.38(18) $Å^3$	
Z	2	
Density (calculated)	1.339 Mg/m^3	
Absorption coefficient	1.059 mm^{-1}	
F(000)	1168	
Crystal habitus	irregular (red)	
Crystal size	0.389 x 0.262 x 0.153 mm^3	
Theta range for data collection	2.220 to 28.282°	
Index ranges	-16<=h<=16, -17<=k<=17, -26<=l<=26	
Reflections collected	140997	
Independent reflections	13820 [R(int) = 0.0447]	
Completeness to θ = 25.242°	99.9 %	
Absorption correction	Gaussian	
Max. and min. transmission	0.975 and 0.948	
Refinement method	Full-matrix least-squares on F^2	
Data / restraints / parameters	13820 / 18 / 641	
Goodness-of-fit on F^2	1.025	
Final R indices [I>2σ(I)]	R1 = 0.0389, wR2 = 0.0950	
R indices (all data)	R1 = 0.0507, wR2 = 0.1013	
Largest diff. peak and hole	1.001 and -0.969 e·$Å^{-3}$	
Crystallisation Details:	CH_2Cl_2/*n*-hexaner.t	

Measurement and Refinement Details: Two disordered CH_2Cl_2 molecules were refined as such.

Single crystal X-ray details of [(IMes)AsP(IDipp)] (4): Crystal Structure of of 4

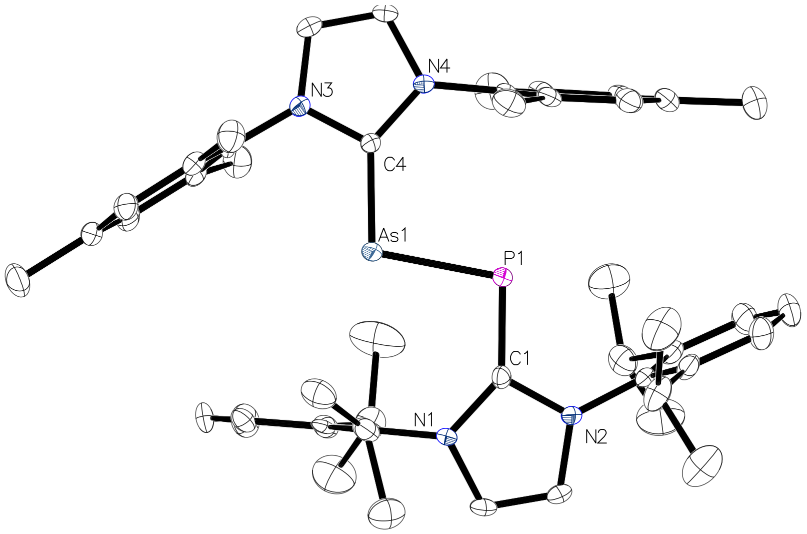

Figure S21. The asymmetric unit of the crystal structure of [(IMes)AsP(IDipp)] (**4**). Hydrogen atoms are omitted for clarity.

CCDC Number	1940132	
Empirical formula	$C_{48}H_{60}AsN_4P$	
Formula weight	798.89	
Temperature	100(2) K	
Wavelength	0.71073 Å	
Instrument (scan mode)	Oxford Diffraction Xcalibur, Eos (ω scan)	
Crystal system	Triclinic	
Space group	$P\bar{1}$	
Unit cell dimensions	a = 10.8463(14) Å	α = 100.194(6)°
	b = 12.9110(8) Å	β = 105.169(10)°
	c = 18.3569(14) Å	γ = 109.671(10)°
Volume	2234.7(4) $Å^3$	
Z	2	
Density (calculated)	1.187 Mg/m^3	
Absorption coefficient	0.832 mm^{-1}	
F(000)	848	
Crystal habitus	plate (red)	
Crystal size	0.251 x 0.197 x 0.095 mm^3	
Theta range for data collection	2.342 to 25.348°	
Index ranges	-13<=h<=13, -15<=k<=15, -22<=l<=22	
Reflections collected	15021	
Independent reflections	15021 [R(int) = 0.1076]	
Completeness to θ = 25.242°	99.9 %	
Absorption correction	Semi-empirical from equivalents	
Max. and min. transmission	1.00000 and 0.98930	
Refinement method	Full-matrix least-squares on F^2	
Data / restraints / parameters	15021 / 0 / 502	
Goodness-of-fit on F^2	0.919	
Final R indices [I>2σ(I)]	R1 = 0.0368, wR2 = 0.0690	
R indices (all data)	R1 = 0.0597, wR2 = 0.0734	
Largest diff. peak and hole	0.825 and -0.516 e·$Å^{-3}$	
Crystallisation Details:	from *n*-hexane at -35°C	

Measurement and Refinement Details: The structure was refined as a two-component twin with component two rotated by -180° around [1.00 -0.00 -0.00] (reciprocal) or [0.93 0.31 0.18] (direct). The relation of the fractional volume contributions of the twin components are 35 % and 65% respectively.

Single crystal X-ray details of [(IMes)AsP(IDipp)]PF6 [5]PF6: Crystal Structure of [(IMes)AsP(IDipp)][PF6]·CH2Cl2 (5[PF6]·CH2Cl2)

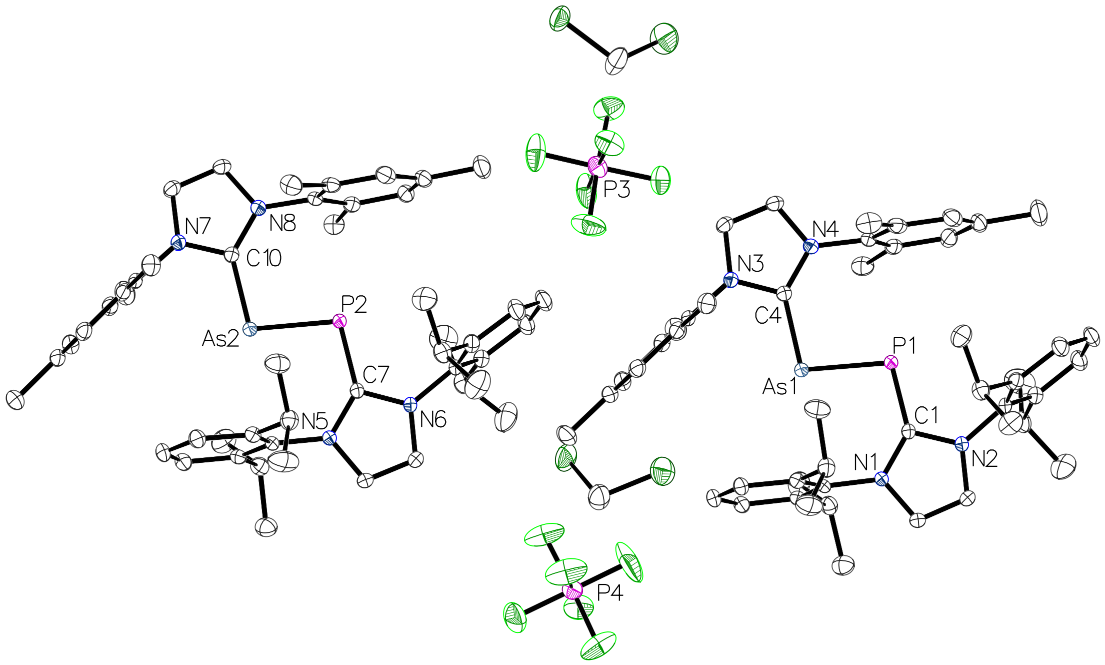

Figure S22. The asymmetric unit of the crystal structure of [(IMes)AsP(IDipp)]PF_6·CH_2Cl_2 ([**5**]PF_6·CH_2Cl_2). Hydrogen atoms are omitted for clarity.

CCDC number	1940133	
Empirical formula	$C_{49}H_{62}AsCl_2F_6N_4P_2$	
Formula weight	1028.78	
Temperature	100(2) K	
Wavelength	1.54184 Å	
Instrument (scan mode)	Oxford Diffraction Xcalibur, Atlas, Nova (ω scan)	
Crystal system	Triclinic	
Space group	$P\bar{1}$	
Unit cell dimensions	a = 17.2209(4) Å	α = 66.609(2)°
	b = 17.5574(4) Å	β = 71.860(2)°
	c = 19.5780(4) Å	γ = 72.677(2)°
Volume	5059.0(2) Å^3	
Z	4	
Density (calculated)	1.351 Mg/m^3	
Absorption coefficient	2.977 mm^{-1}	
F(000)	2140	
Crystal habitus	block (green)	
Crystal size	0.177 x 0.159 x 0.111 mm^3	
Theta range for data collection	3.300 to 76.240°	
Index ranges	-21<=h<=21, -22<=k<=22, -24<=l<=24	
Reflections collected	240672	
Independent reflections	21126 [R(int) = 0.0437]	
Completeness to θ = 67.684°	100.0 %	
Absorption correction	Gaussian	
Max. and min. transmission	0.982 and 0.971	
Refinement method	Full-matrix least-squares on F^2	
Data / restraints / parameters	21126 / 0 / 1181	
Goodness-of-fit on F^2	1.020	
Final R indices [I>2σ(I)]	R1 = 0.0349, wR2 = 0.0943	
R indices (all data)	R1 = 0.0377, wR2 = 0.0968	
Largest diff. peak and hole	1.150 and -0.722 e·Å^{-3}	
Crystallisation Details:	CH_2Cl_2/*n*-hexane	

Single crystal X-ray details of dicationic compound [(IMes)AsP(IDipp)][$GaCl_4$]$_2$ ([6][$GaCl_4$]$_2$): Crystal Structure of [(IMes)AsP(IDipp)][$GaCl_4$]·2C_6H_5F (6[$GaCl_4$]·2C_6H_5F)

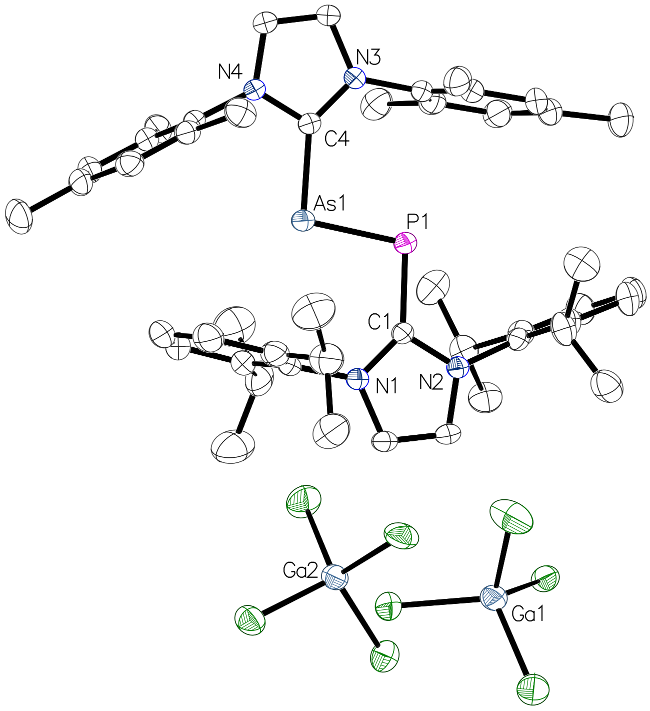

Figure S23. The asymmetric unit of the crystal structure of [(IMes)AsP(IDipp)][$GaCl_4$]$_2$·2C_6H_5F ([**6**][$GaCl_4$]$_2$·2C_6H_5F). Hydrogen atoms and two molecules of fluorobenzene are omitted for clarity.

CCDC number	1940134	
Empirical formula	$C_{60}H_{70}AsCl_8F_2Ga_2N_4P$	
Formula weight	1414.13	
Temperature	100(2) K	
Wavelength	1.54184 Å	
Instrument (scan mode)	Oxford Diffraction Xcalibur, Atlas, Nova (ω scan)	
Crystal system	Triclinic	
Space group	$P\bar{1}$	
Unit cell dimensions	a = 12.5745(4) Å	α = 98.702(2)°
	b = 13.1188(4) Å	β = 90.233(2)°
	c = 22.3946(6) Å	γ = 113.079(2)°
Volume	3351.30(18) $Å^3$	
Z	2	
Density (calculated)	1.401 Mg/m^3	
Absorption coefficient	5.049 mm^{-1}	
F(000)	1444	
Crystal habitus	irregular (orange)	
Crystal size	0.285 x 0.223 x 0.111 mm^3	
Theta range for data collection	3.714 to 76.341°	
Index ranges	-15<=h<=15, -16<=k<=16, -28<=l<=28	
Reflections collected	138873	
Independent reflections	13977 [R(int) = 0.0600]	
Completeness to θ = 67.684°	99.9 %	
Absorption correction	Gaussian	
Max. and min. transmission	0.984 and 0.961	
Refinement method	Full-matrix least-squares on F^2	
Data / restraints / parameters	13977 / 0 / 717	
Goodness-of-fit on F^2	1.028	
Final R indices [I>2σ(I)]	R1 = 0.0507, wR2 = 0.1359	
R indices (all data)	R1 = 0.0559, wR2 = 0.1408	
Largest diff. peak and hole	2.727 and -1.213 e·$Å^{-3}$	
Crystallisation Details:	from fluorobenzene	

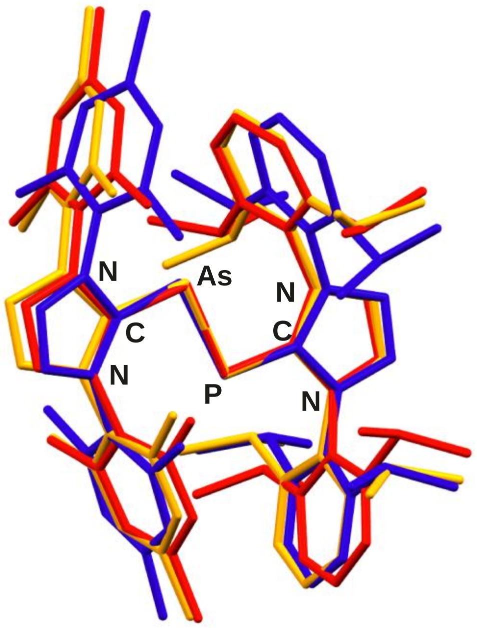

Figure S24. Overlay of the molecular structures of **4** (orange), $\mathbf{5}^{+\bullet}$ (blue) and $\mathbf{6}^{2+}$ (red).

D) Computational details

All computations were performed using the density functional method B97-D (S. Grimme) as implemented in the Gaussian09 program.[8] For all main group elements (C, H, P and As) the all-electron triple-ζ basis set (6-311G**) was used.[9] Natural Bond Orbital (NBO) analysis (NBO charges, WBI) was carried out using NBO version 3,[10] which is part of the Gaussian09 program package. For quantitative analysis of the spin density the free software Muliwfn 3.6 was used.[11]

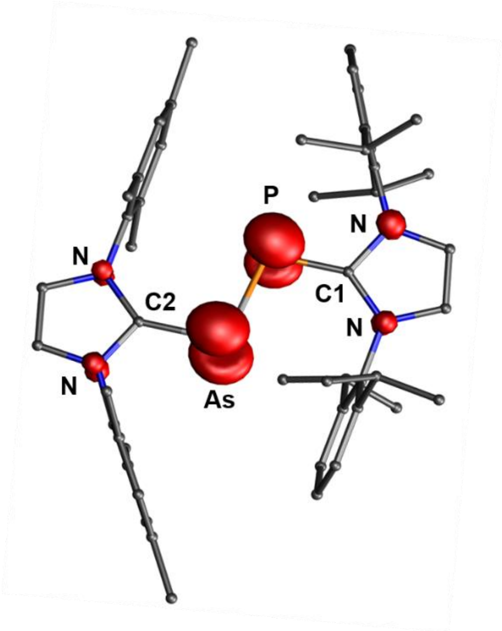

Figure S25. Spin density distribution of the radical cation in [**5**]PF_6.

Table S1. Energies for all optimized structures.

**Compound**	E_{0K}[a] [Ha]	E_{298K}[b] [Ha]	H_{298K}[b] [Ha]	G_{298K}[b] [Ha]
**4**	-4660.693387	-4660.633524	-4660.632580	-4660.792051
**5**$^{+\bullet}$	-4660.540432	-4660.481201	-4660.480257	-4660.638026
**6**$^{2+}$	-4660.257946	-4660.198661	-4660.197717	-4660.354039

[a] DFT energy incl. ZPE.
[b] standard conditions T = 298.15 K and p = 1 atm.

Table S2. Selected bond lengths and bond angles computed in **4**, **5**$^{+\bullet}$ and **6**$^{2+}$.

Bond distance [Å]	**4**	**5**$^{+\bullet}$	**6**$^{2+}$
C6-As114	1.908	1.957	1.987
As114-P113	2.356	2.288	2.216
P113-C1	1.772	1.810	1.837
Bond angle [°]			
∠(C6-As114-P113)	100.7	98.8	97.4
∠(As114-P113-C1)	102.4	100.9	101.1
∠(Im_{Mes}-Im_{Dipp})	29.2	10.8	23.3
Torsion angle [°]			
∠(C_{Mes}-As-P-C_{Dipp})	-152.0	-175.0	-175.1
∠(N111-C6-As114-P113)	173.0	173.2	161.5
∠(N110-C1-P113-As114)	-169.2	-168.1	175.8

Table S3. Energies for all optimized structures.

Atom	Spin density value	% of Sum
109(N)	0.04	4.1
110(N)	0.05	4.9
111(N)	0.04	4.0
112(N)	0.03	3.5
113(P)	0.31	31.1
114(As)	0.39	38.4

E) References

1) A. Doddi, D. Bockfeld, T. Bannenberg, P. G. Jones, M. Tamm, *Angew. Chem. Int. Ed.* **2014**, *53*, 13568.
2) M. Y. Abraham, Y. Wang, Y. Xie, P. Wei, H. F. Schaefer, Schleyer, P von R, G. H. Robinson, *Chem. Eur. J.* **2010**, *16*, 432.
3) S. Stoll and A. Schweiger, *J. Magn. Reson*., **2006**, *178*, 42.
4) Rigaku Oxford Diffraction, CrysAlisPRO Software System, version 1.171.38.43 (**2015**), Rigaku Corporation, Oxford, UK.
5) G. M. Sheldrick, *Acta Cryst*. **2015**, *A71*, 3-8.
6) G. M. Sheldrick, *Acta Cryst*. **2015**, *C71*, 3-8.
7) L. J. Farrugia, *J. Appl. Cryst*. **2012**, *45*, 849-854.
8) Gaussian 09, Revision A.1, M. J. Frisch, G. W. Trucks, H. B. Schlegel, G. E. Scuseria, M. A. Robb, J. R. Cheeseman, G. Scalmani, V. Barone, B. Mennucci, G. A. Petersson, H. Nakatsuji, M. Caricato, X. Li, H. P. Hratchian, A. F. Izmaylov, J. Bloino, G. Zheng, J. L. Sonnenberg, M. Hada, M. Ehara, K. Toyota, R. Fukuda, J. Hasegawa, M. Ishida, T. Nakajima, Y. Honda, O. Kitao, H. Nakai, T. Vreven, J. A. Montgomery, Jr., J. E. Peralta, F. Ogliaro, M. Bearpark, J. J. Heyd, E. Brothers, K. N. Kudin, V. N. Staroverov, R. Kobayashi, J. Normand, K. Raghavachari, A. Rendell, J. C. Burant, S. S. Iyengar, J. Tomasi, M. Cossi, N. Rega, J. M. Millam, M. Klene, J. E. Knox, J. B. Cross, V. Bakken, C. Adamo, J. Jaramillo, R. Gomperts, R. E. Stratmann, O. Yazyev, A. J. Austin, R. Cammi, C. Pomelli, J. W. Ochterski, R. L. Martin, K. Morokuma, V. G. Zakrzewski, G. A. Voth, P. Salvador, J. J. Dannenberg, S. Dapprich, A. D. Daniels, Ö. Farkas, J. B. Foresman, J. V. Ortiz, J. Cioslowski, and D. J. Fox, Gaussian, Inc., Wallingford CT, **2009**.
9) X. Cao, M. Dolg, *J. Chem. Phys.*, **2001**, *115*, 7348.
10) a) J. P. Foster and F. Weinhold, *J. Am. Chem. Soc.*, **1980**, *102*, 7211; b) A. E. Reed, F. Weinhold, *J. Chem. Phys.*, **1983**, *78*, 4066; c) A. E. Reed, R. B. Weinstock, F. Weinhold, *J. Chem. Phys.*, **1985**, *83*, 735; d) A. E. Reed, F. Weinhold, *J. Chem. Phys.*, **1985**, *83*, 1736.
11) T. Lu, Multiwfn (Version 3.6) - A Multifunctional Wave Function Analyzer, http://sobereva.com/multiwfn/
